# Engineering a Gram-Negative Bactericidal Hydrogel: Cu_x_Te Nanozyme Functions as a Specific Killer by Hijacking LPS and Flagella Biosynthesis

**DOI:** 10.34133/research.1140

**Published:** 2026-02-25

**Authors:** Jianguo Niu, Yuhao Xue, Wenqi Wang, Wei Zhang, Min Wang, Jiaqi Qin, Dongliang Yang, Xianwen Wang

**Affiliations:** ^1^ School of Biomedical Engineering, Anhui Medical University, Hefei 230022, P. R. China.; ^2^ School of Physical and Mathematical Sciences, Nanjing Tech University, Nanjing 211816, P. R. China.

## Abstract

The treatment of gram-negative bacterial infections remains a formidable challenge due to their resilient outer membrane and adaptive evasion mechanisms. Herein, we present a multifunctional nanozyme hydrogel, copper telluride@cationic guar gum (Cu_x_Te@CG), which acts as a gram-negative-specific bactericidal platform. This hydrogel integrates the unique enzymatic and physical properties of urchin-like Cu_x_Te nanozymes with the biocompatible and adhesive cationic guar gum (CG) matrix. The Cu_x_Te@CG hydrogel exhibits synergistic oxidase- and glutathione peroxidase-like activities, catalyzing the generation of reactive oxygen species (ROS) while depleting bacterial glutathione, thereby inducing lethal oxidative stress. Crucially, transcriptome sequencing revealed that the platform specifically targets *Pseudomonas aeruginosa* by down-regulating key genes involved in lipopolysaccharide (LPS) biosynthesis and flagellar assembly, compromising their primary defense and motility structures. This targeted interference with LPS and flagella amplifies the ROS-mediated attack, leading to enhanced and specific killing of gram-negative pathogens (*Escherichia coli*, *P. aeruginosa*, and *Klebsiella pneumoniae*), effective biofilm disruption, and inhibition. In a *P. aeruginosa*-infected burn wound model, the Cu_x_Te@CG hydrogel markedly accelerated healing by eliminating bacteria, promoting angiogenesis, and modulating inflammation, all while demonstrating excellent biosafety. This work establishes the Cu_x_Te@CG hydrogel as a robust and targeted therapeutic strategy for combating stubborn gram-negative infections.

## Introduction

Gram-negative bacteria such as *Pseudomonas aeruginosa*, *Klebsiella pneumoniae* (*K. pneumoniae*), *Escherichia coli* (*E. coli*), and *Acinetobacter baumannii* (*A. baumannii*) have developed resistance to most antibiotics currently used in clinical practice and, in some cases, all antibiotics [1]. Gram-negative bacteria are notorious for their ability to reduce the barrier property of the bacterial outer membrane by regulating the formation of lipopolysaccharide (LPS), thereby preventing the effective penetration of antimicrobial agents [2]. Furthermore, gram-negative bacteria utilize flagella to increase their ability to colonize hosts and evade antimicrobial attacks [3]. Moreover, gram-negative bacteria are highly prone to forming biofilms, which are equipped with a dense extracellular polymeric substance (EPS) barrier and a biofilm microenvironment (BME), effectively protecting the bacteria [4]. Consequently, the difficulty in developing effective antibacterial agents against gram-negative bacteria has become a marked factor in the current crisis of bacterial resistance [5,6]. Gram-negative bacterial infections are extremely common in burn wounds, with *P. aeruginosa* accounting for up to 57.45% of infections in severely burned patients [7]. Owing to their characteristics as gram-negative bacteria, these bacteria are capable of producing multiple drug resistance mechanisms to combat existing antibiotic treatments. Currently, the clinical treatment of burn wounds infected with *P. aeruginosa*, a gram-negative bacterium, faces notable challenges [8].

The advancement of nanozymes has offered promise for the treatment of drug-resistant bacterial infections [9]. Copper-based nanozymes, as widely adopted nanozymes, present outstanding physical and chemical attributes and are broadly utilized in wound treatment [10]. In comparison to traditional antibiotic drugs, copper-based nanozymes can efficiently break down bacterial structure and metabolism to overcome bacterial drug resistance by releasing copper ions, catalyzing the generation of reactive oxygen species (ROS), or directly physically contacting the bacterial cell wall [11–13]. These multimodal antimicrobial mechanisms endow copper-based nanozymes with efficient and broad-spectrum bactericidal effects, but their indiscriminate bactericidal ability can also cause cytotoxicity and microecological imbalance at the site of the lesion, which can result in harmful consequences on the body, such as inflammation and immune dysregulation [14,15]. Therefore, how to selectively inhibit target bacteria via copper-based nanozymes is a current research challenge. Copper-based nanozymes have been designed to selectively kill gram-positive bacteria [16–18]. Nevertheless, owing to the complex structure of LPS (a conserved phosphoglycolipid [lipid A], a relatively short oligosaccharide chain [core OS region], and a surface-exposed O-polysaccharide [O-antigen, O-specific chain]) and the ability of flagella to benefit and avoid harm, few reports of copper-based nanozymes that selectively kill gram-negative bacteria exist [19–22]. Currently, researchers have designed nanomaterials that selectively target gram-negative bacteria, such as “peptide photosensitizer conjugates enhanced photodynamic therapy through membrane disruption of gram-negative bacteria” [23] and “microwave-assisted garcinia nanoparticles for antibacterial effects against gram-negative bacteria” [24]. However, the complexity of applying these antibacterial strategies limits their prospects for practical use. Therefore, to overcome the above issues and challenges, further development and research are needed for therapeutic strategies that target and kill gram-negative bacteria.

**Fig. 1.**
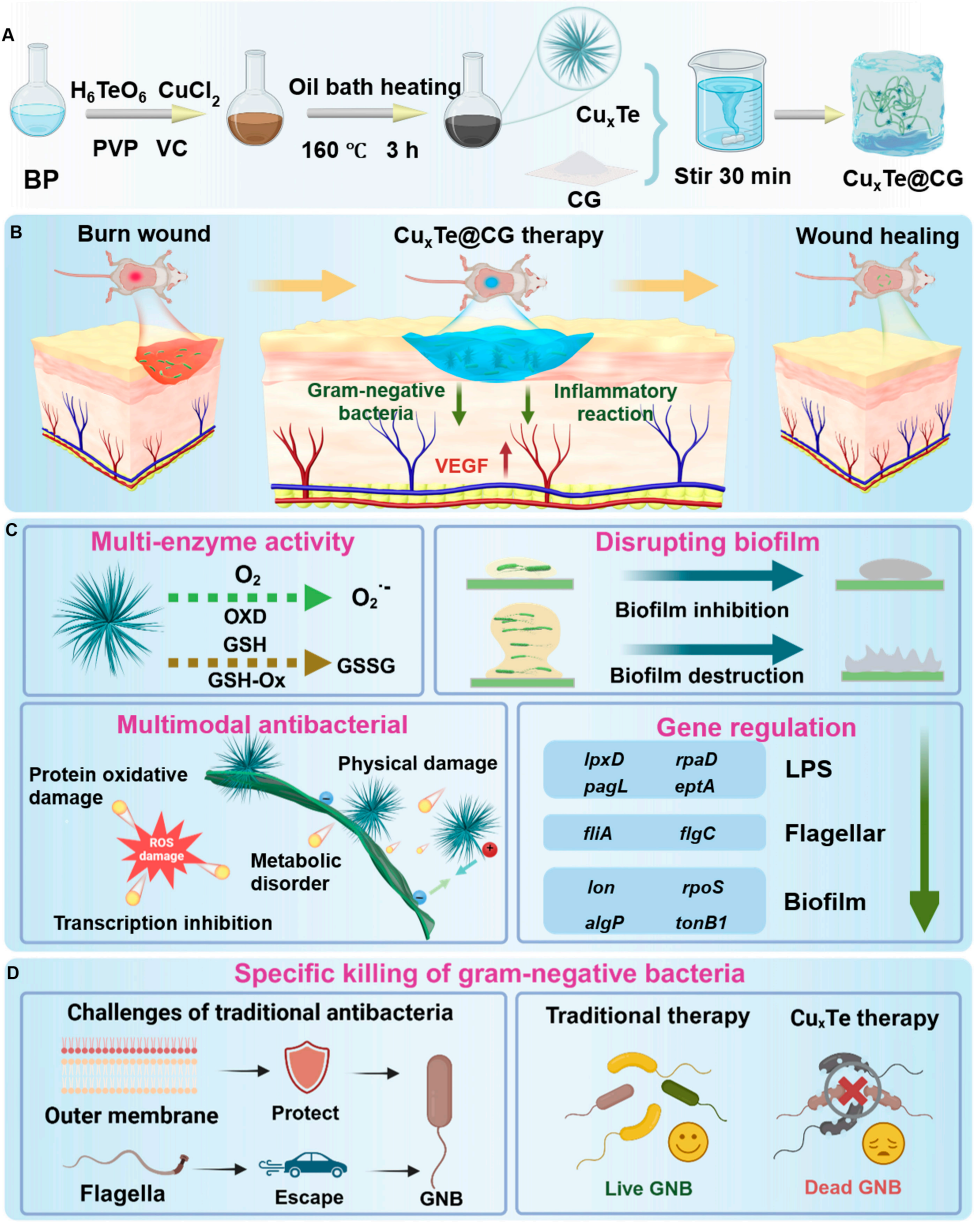
Schematic illustration depicting the synthesis process and antibacterial application of the Cu_x_Te@CG hydrogel. (A) A schematic representation of the preparation of the Cu_x_Te@CG hydrogel through the loading of Cu_x_Te nanozymes onto CG via a wet chemical method. (B) Schematic diagram illustrating the enhancement of burn wound healing in *P. aeruginosa*-infected wounds by the Cu_x_Te@CG hydrogel. (C) Schematic diagram of the antibacterial mechanism of Cu_x_Te nanozymes. (D) Schematic illustration of the specific killing effect of the Cu_x_Te@CG hydrogel on gram-negative bacteria (GNB). Created in BioRender.

To overcome these challenges, this study successfully developed an urchin-like Cu_x_Te nanozymes integrated into a cationic guar gum hydrogel, forming the multifunctional Cu_x_Te@CG platform (Fig. 1A), this system orchestrates a gram-negative-specific antibacterial strategy through a combination of physical and enzymatic actions (Fig. 1B to D). The positively charged, spiky architecture of Cu_x_Te nanozymes enables effective targeting and initial physical disruption of bacterial membranes. Furthermore, its urchin-like morphology provides abundant catalytic sites, enhancing its oxidase- and glutathione peroxidase-like activities. This dual enzyme mimicry not only catalyzes the generation of bactericidal superoxide anions but also depletes bacterial glutathione (GSH), thereby augmenting oxidative stress. Crucially, our investigation revealed that the exceptional specificity of the Cu_x_Te@CG hydrogel stems from its ability to interfere with the biosynthesis of LPSs and flagella, structures quintessential to gram-negative bacterial defense and virulence. This multifaceted mechanism, which physically damages, chemically attacks, and genetically weakens the target pathogens, demonstrates remarkable efficacy in treating *P. aeruginosa*-infected burn wounds.

## Results and Discussion

### Synthesis, characterization, and enzyme-like activity detection of Cu_x_Te nanozymes

**Fig. 2.**
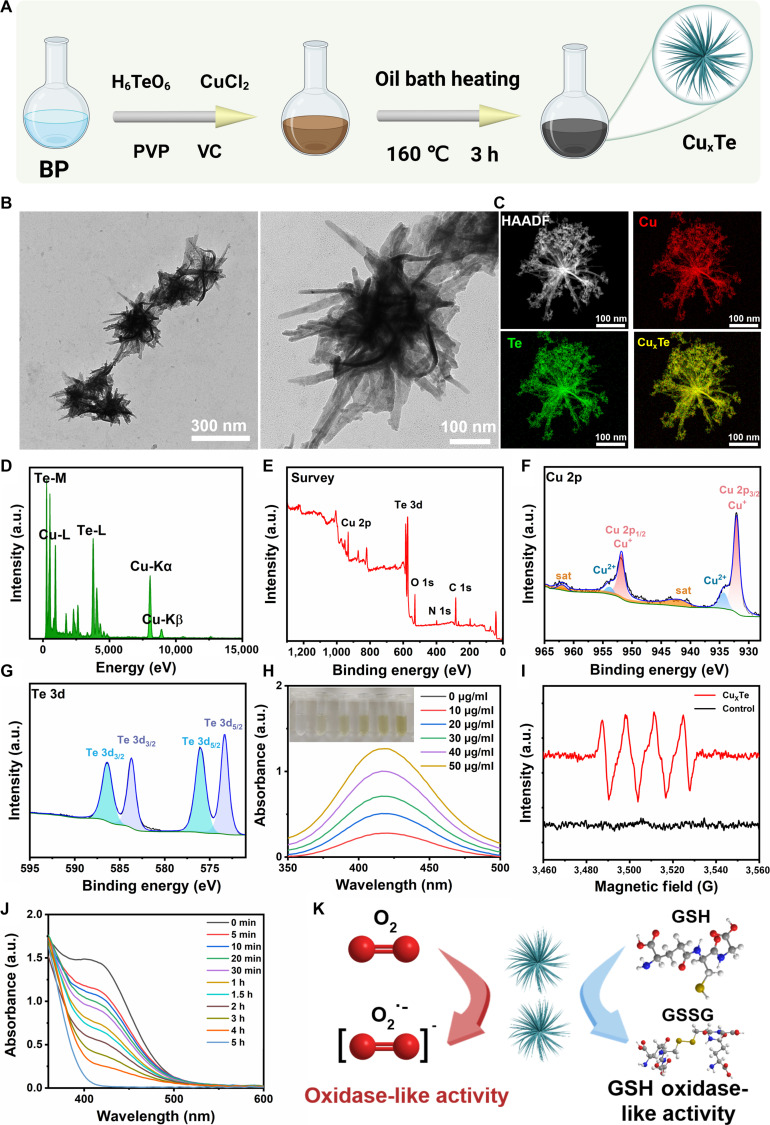
Synthesis, characterization, and enzyme-like activity detection of Cu_x_Te nanozymes. (A) Schematic diagram of the principle of synthesis (created in BioRender). (B) TEM images of Cu_x_Te nanozymes. (C) HAADF-STEM image and elemental mappings. (D) EDS of Cu_x_Te nanozymes. (E to G) XPS survey of Cu_x_Te nanozymes, as well as the XPS spectra of Cu 2p and Te 3d. (H and I) OXD-like activity of Cu_x_Te nanozymes (50 μg/ml) using an OPD probe and ESR. (J) Time-dependent GSH depletion of Cu_x_Te nanozymes (50 μg/ml) at the same concentrations using the DTNB probe. (K) Schematic diagram of Cu_x_Te nanozymes with OXD-like and GSH-Ox-like activities.

Cu_x_Te nanozymes with an urchin-like morphology were synthesized in one step via a wet chemical method, with copper chloride and telluric acid as reaction precursors, benzyl alcohol as the reaction solvent, ascorbic acid as the reducing agent, and polyvinylpyrrolidone (PVP) as an auxiliary (Fig. 2A). Transmission electron microscopy (TEM) captured the urchin-like microstructure of Cu_x_Te nanozymes (Fig. 2B). As shown in the high-resolution transition electron microscopy (HRTEM) image, Cu_x_Te nanozymes had a large specific surface area and sharp spikes, presenting an urchin-like morphology, and the elemental mapping results indicated that the elements Cu (red) and Te (green) were evenly distributed on the surface of Cu_x_Te nanozymes, demonstrating the successful construction of a 3-dimensional (3D) urchin-like nanostructure (Fig. 2C). The energy-dispersive x-ray spectroscopy (EDS) results (Fig. 2D) further revealed the elemental and chemical compositions of the samples. The x-ray diffraction (XRD) patterns of Cu_x_Te nanozymes were investigated in the range of 10° to 80° (2θ), and the crystal structure and properties were characterized (Fig. S1). The XRD data revealed diffraction peaks at 2θ values of 25.26°, 30.78°, 31.15°, 42.18°, 44.67°, and 45.91°, which were attributed to the (011), (011), (101), (012), (112), and (110) facets of the spinel structure, respectively, in agreement with those of CuTe (PDF#04-008-8274) and Cu_2_Te (PDF#04-007-6583). Additionally, x-ray photoelectron spectroscopy (XPS) was employed to determine the chemical valence states of Cu and Te, thereby providing further insights into the elemental composition and bonding states (Fig. 2E). The peaks at 951.8 and 932 eV correspond to the Cu^+^ 2p_3/2_ and 2p_1/2_ orbitals, respectively, whereas those at 953.8 and 934.2 eV correspond to the Cu^2+^ 2p_3/2_ and 2p_1/2_ orbitals accompanied by 2 satellite peaks of Cu^2+^ (Fig. 2F). Thus, the XPS spectrum of Cu 2p indicated the presence of Cu in a mixed valence state of Cu^2+^ and Cu^+^ within Cu_x_Te nanozymes. Regarding the molar ratio of Cu^+^ to Cu^2+^, it was determined to be 5.62:1 through quantitative calculation of peak areas and molar ratio conversion from the high-resolution XPS spectrum of Cu 2p_3/2_. Based on the distinct electronic configurations and catalytic roles of Cu^+^ and Cu^2+^ in multivalent copper nanozymes, it can be assumed that Cu^2+^ is the relatively “dormant” valence state, while Cu^+^ is the active state. The peaks at 583.7 and 573.3 eV correspond to the Te^2−^ 3d_3/2_ and 3d_5/2_ orbitals, respectively, whereas those at 586.4 and 576 eV correspond to the Te^4+^ 3d_3/2_ and 3d_5/2_ orbitals, respectively (Fig. 2G). Therefore, the XPS spectrum of Te 3d demonstrated that Te exists in a mixed valence state of Te^2−^ and Te^4+^ within Cu_x_Te nanozymes. The measured average zeta potential was +13.57 mV, indicating that Cu_x_Te nanozymes had a positive charge and could electrostatically attract negatively charged bacteria (Fig. S2). Cu_x_Te nanozymes measured a particle size of 316 nm, which matches the size determined via TEM (Fig. S3). Experiments on ROS generation and enzyme-like activity detection effectively reflected the oxidative damage characteristics of nanomaterials to bacteria. Cu_x_Te nanozymes could directly react with the o-phenylenediamine (OPD) probe under acidic conditions (Fig. S4) in proportion to the concentration (Fig. 2H), suggesting that Cu_x_Te nanozymes were capable of generating superoxide anions, which oxidize OPD to a yellow product with an absorption peak at 442 nm. These findings suggested that Cu_x_Te nanozymes possessed OXD-like enzyme activity. To further confirm that Cu_x_Te nanozymes have OXD-like enzyme activity, 5,5-dimethyl-1-pyrroline N-oxide (DMPO) was utilized as a radical scavenger, and the characteristic peak of superoxide anions was evaluated via electron spin resonance (ESR) (Fig. 2I) [25]. Moreover, the results of the GSH consumption experiment demonstrated that Cu_x_Te nanozymes had a strong ability to consume reducing substrates (Fig. 2J and Fig. S5). Therefore, the ability of Cu_x_Te nanozymes to consume GSH could enhance their oxidative performance during antibacterial activity, thereby achieving better bactericidal effects. The proposed mechanism illustrates how Cu_x_Te nanozymes generate superoxide radicals while simultaneously demonstrating OXD-like and GSH-Ox-like enzymatic activities (Fig. 2K). Cu_x_Te nanozymes exhibited favorable stability in aqueous solution. TEM characterization on day 1 and day 10 post-synthesis showed no obvious structural destruction or morphological alteration, and OPD/5,5′-dithiobis (2-nitrobenzoic acid) (DTNB)-based catalytic performance tests presented no significant differences at the 2 time points (Fig. S6). Notably, the copper ion release behavior of Cu_x_Te nanozymes is closely related to their antibacterial performance and environmental stability. Our detection results of the copper ion release profile in phosphate-buffered saline (PBS) at different pH values showed that only a small amount of copper ions were released at pH 7.2, an observation that indirectly verifies the good stability of Cu_x_Te nanozymes in neutral aqueous systems. In sharp contrast, the copper ion release levels at pH 6.0 and 5.0 were 4.11-fold and 9.45-fold higher than those at pH 7.2, respectively. Considering that bacterial infection usually leads to an acidic microenvironment via metabolic products, the pH-dependent copper ion release of Cu_x_Te nanozymes enables them to release copper ions in infection sites, which further synergizes with other antibacterial mechanisms to realize excellent bactericidal efficacy (Fig. S7).

### In vitro antibacterial performance of Cu_x_Te nanozymes

**Fig. 3.**
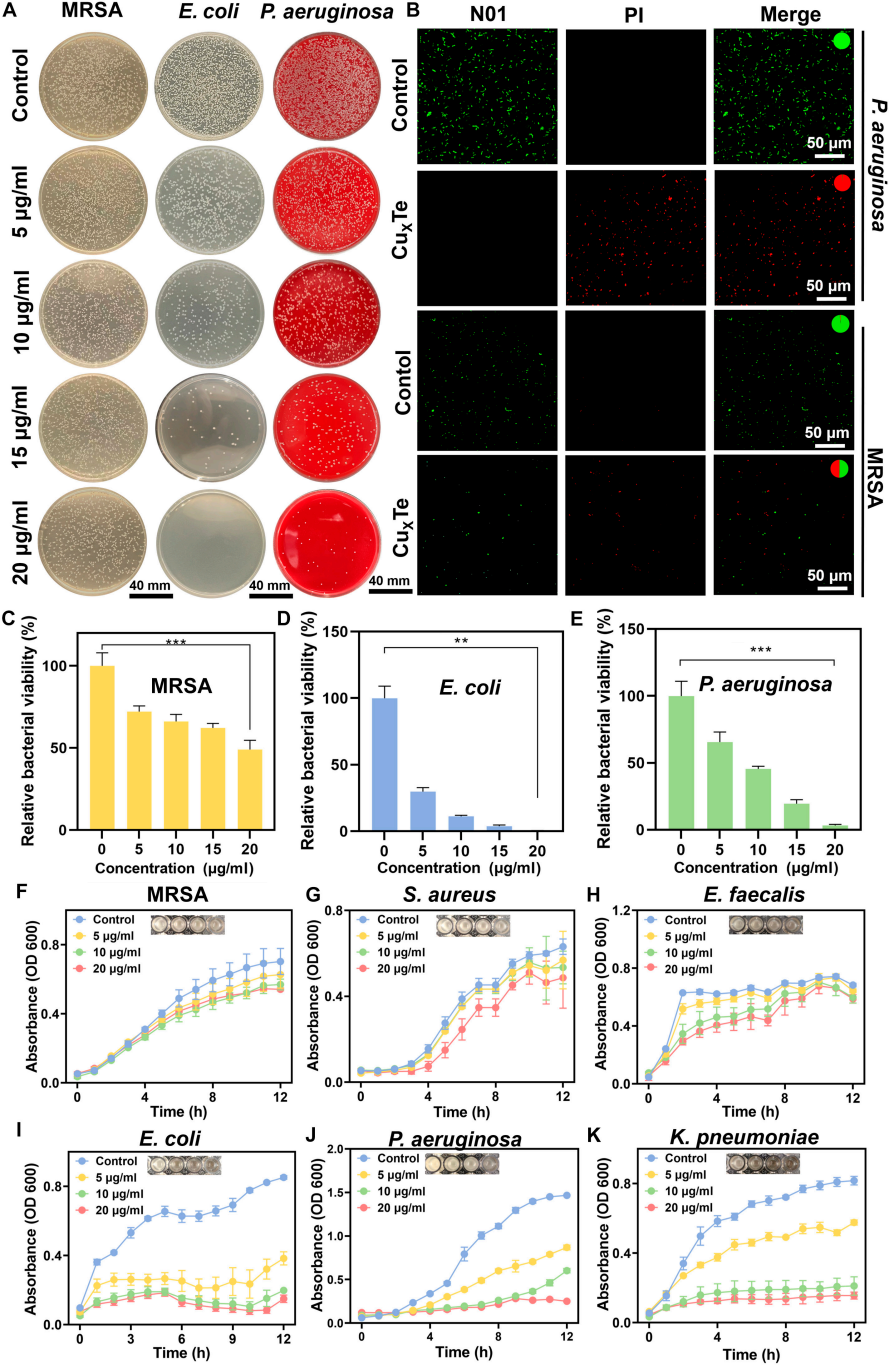
In vitro antibacterial performance of Cu_x_Te nanozymes. (A) Photographs of MRSA, *E. coli*, and *P. aeruginosa* bacterial colonies. (B) Fluorescence images of dead (red) and live (green) bacteria of *P. aeruginosa* and MRSA in Cu_x_Te nanozymes (20 μg/ml) and the control group. (C to E) Relative bacterial viability of MRSA, *E. coli*, and *P. aeruginosa* under different conditions. (F to H) Growth curves of MRSA, *S. aureus*, and *E. faecalis* at different concentrations of Cu_x_Te nanozymes. (I to K) Growth curves of *E. coli*, *P. aeruginosa*, and *K. pneumoniae* at different concentrations of Cu_x_Te nanozymes. Statistical analysis was performed using one-way ANOVA followed by Tukey’s post hoc test (**P < 0.01 and ***P < 0.001) (mean ± SD, *n* = 3).

Cu_x_Te nanozymes possess an urchin-like morphology with positive charges that can target negatively charged bacteria. Additionally, Cu_x_Te nanozymes could generate ROS and deplete GSH. In addition, tellurium-based nanomaterials exhibit superior effects against gram-negative bacteria [26,27]. Therefore, tellurium-based nanozymes may have a more efficient antibacterial effect on gram-negative bacteria. Bacterial dilution and plating experiments demonstrated that, compared with methicillin-resistant *Staphylococcus aureus* (MRSA), Cu_x_Te nanozymes exhibited significantly better antibacterial effects against *E. coli* and *P. aeruginosa*, which are representative gram-negative bacteria (Fig. 3A). The relative bacterial viabilities of MRSA, *E. coli*, and *P. aeruginosa* at a Cu_x_Te concentration of 20 μg/ml were 49%, 3.8%, and 1.7%, respectively, highlighting their specific effects on gram-negative bacteria at very low concentrations (Fig. 3C to E). Furthermore, bacteria were stained with PI (red fluorescence stain for dead bacteria) and N01 (green fluorescence stain for live bacteria) to observe their viability status, further confirming the superior antibacterial ability of Cu_x_Te nanozymes against *E. coli* and *P. aeruginosa* compared with that of MRSA (Fig. 3B and Fig. S8). To further validate the specific targeting and killing effects of Cu_x_Te nanozymes on gram-negative bacteria, the growth curves of 3 gram-positive bacteria and 3 gram-negative bacteria were measured. At a concentration of 20 μg/ml, the ability of Cu_x_Te nanozymes to inhibit the growth of the gram-positive bacteria MRSA, *S. aureus*, and *Enterococcus faecalis* (Fig. 3F to H) was significantly weaker than that of the gram-negative bacteria *E. coli*, *P. aeruginosa*, and *K. pneumoniae* (Fig. 3I to K).

### Antibacterial mechanism of Cu_x_Te nanozymes

**Fig. 4.**
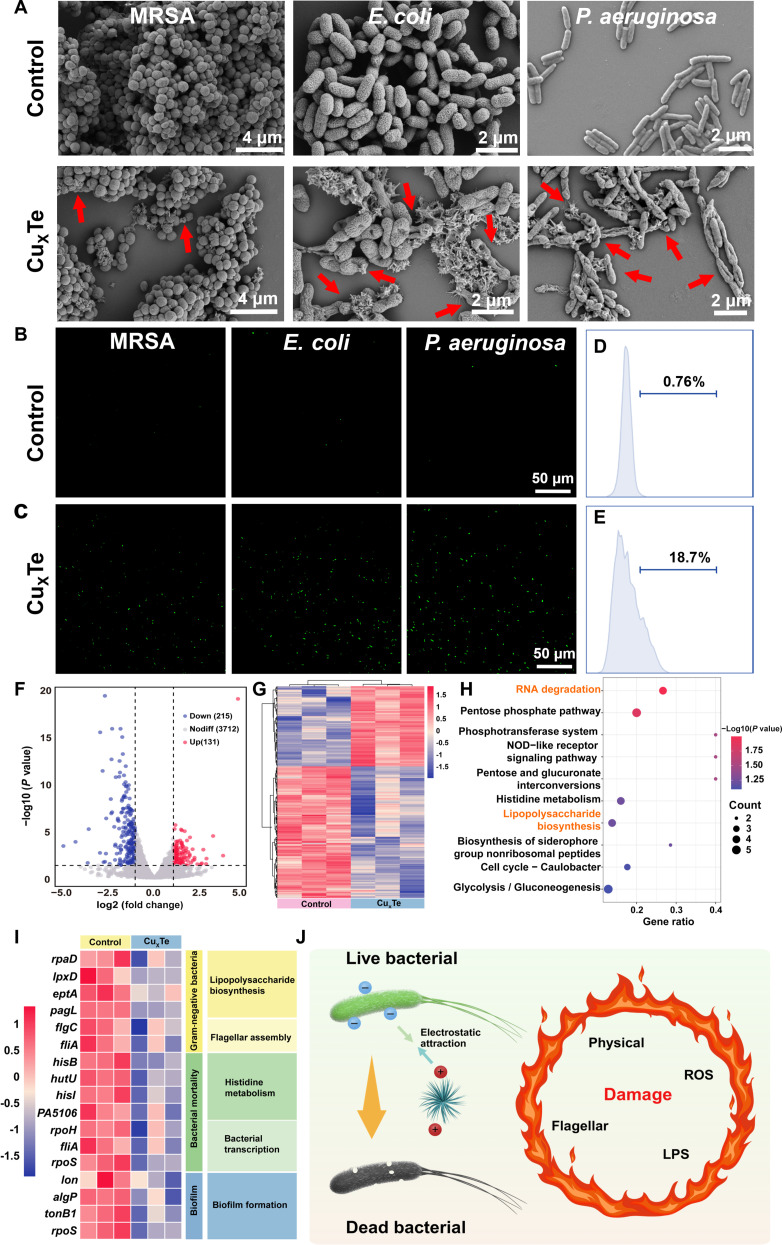
Antibacterial mechanism of Cu_x_Te nanozymes. (A) SEM images of MRSA, *E. coli*, and *P. aeruginosa* in Cu_x_Te nanozymes (20 μg/ml) and the control group. (B and C) Fluorescence images of the ROS (green) of the bacteria MRSA, *E. coli*, and *P. aeruginosa* in Cu_x_Te nanozymes (20 μg/ml) and the control group. (D and E) Quantitative analysis of ROS in different *P. aeruginosa* in Cu_x_Te nanozymes (20 μg/ml) and the control group*.* (F) Volcano plot analyses of total DEGs in *P. aeruginosa* after treatment with PBS and Cu_x_Te nanozymes (20 μg/ml). (G) Gene heatmap after treatment with PBS or Cu_x_Te nanozymes. (H) KEGG enrichment analysis. (I) Heatmap of pathway genes with significant impacts. (J) Schematic diagram of the antibacterial mechanism of Cu_x_Te nanozymes (created in BioRender).

In vitro, antibacterial experiments confirmed that Cu_x_Te nanozymes had superior antibacterial activity against gram-negative bacteria. However, the mechanism underlying this specific killing of gram-negative bacteria requires further investigation and exploration. To delve into the antibacterial mechanism of Cu_x_Te nanozymes, first, microscopic observations of MRSA, *E. coli*, and *P. aeruginosa* both untreated and treated with Cu_x_Te nanozymes were conducted by scanning electron microscopy (SEM) (Fig. 4A). The untreated bacteria (control group) did not exhibit any abnormal morphological changes. However, in bacteria treated with Cu_x_Te nanozymes (Cu_x_Te group), MRSA, *E. coli*, and *P. aeruginosa* all displayed holes (indicated by red arrows), and Cu_x_Te nanozymes adhered well to the bacteria (indicated by red arrows), confirming that Cu_x_Te nanozymes could adhere to bacteria through electrostatic attraction and disrupt them with spikes. Notably, the gram-negative bacteria in the Cu_x_Te nanozymes group presented a greater degree of disruption and a greater number of affected bacteria, which aligns with the specific killing of gram-negative bacteria by Cu_x_Te nanozymes. The ROS levels within the bacteria were subsequently measured via a 2′,7′-dichlorodihydrofluorescein (DCFH-DA) probe. Green fluorescent signals were detected in the MRSA, *E. coli*, and *P. aeruginosa* strains that had been treated with Cu_x_Te nanozymes. However, the green fluorescence intensity was significantly greater in the *E. coli* and *P. aeruginosa* strains than in the MRSA strains, suggesting that ROS may be one of the reasons for the specific killing effect of Cu_x_Te nanozymes (Fig. 4B and C). To further demonstrate the impact of the ROS catalyzed by Cu_x_Te nanozymes on *P. aeruginosa*, Cu_x_Te-treated *P. aeruginosa* was stained with the DCFH-DA probe, and the fluorescence intensity was measured using flow cytometry. The ROS in the Cu_x_Te-treated group exhibited a marked and statistically significant increase compared to that in the control group (Fig. 4D and E). To further validate the ROS-mediated antibacterial mechanism and clarify the association between lipid peroxidation and the bactericidal effect of Cu_x_Te nanozymes, malondialdehyde (MDA)—a primary biomarker for evaluating lipid peroxidation—was selected for supplementary detection. MDA levels in 4 bacterial strains (MRSA, *E. coli*, *P. aeruginosa*, and *K. pneumoniae*) were determined using a commercial MDA assay kit. The results showed that regardless of whether the bacteria were gram-positive (MRSA) or gram-negative (*E. coli*, *P. aeruginosa*, *K. pneumoniae*), the MDA content in Cu_x_Te-treated groups was significantly higher than that in the untreated control groups. Notably, the MDA levels in the 3 gram-negative strains were nearly twice as high as those in the gram-positive MRSA. This observation was highly consistent with the ROS level results previously detected by the DCFH-DA probe, further confirming that Cu_x_Te nanozymes exert a specific bactericidal effect on gram-negative bacteria through ROS-induced lipid peroxidation, which deepens the understanding of its antibacterial mechanism (Fig. S9). Consequently, Cu_x_Te nanozymes utilize their physical properties (positive charge and spikes) to cause a certain degree of disruption to bacteria, and in combination with the ROS catalyzed by their multienzyme activity, they have a detrimental effect on bacteria. To further explore the antibacterial mechanism of Cu_x_Te nanozymes, prokaryotic gene transcriptome analysis was conducted on *P. aeruginosa* from both the control group (treated with PBS) and the Cu_x_Te nanozymes group (treated with Cu_x_Te). The sequencing results revealed 346 genes that were differentially expressed between the control and Cu_x_Te groups. Compared with those in the control group, 215 genes were down-regulated, and 131 genes were up-regulated in Cu_x_Te-treated *P. aeruginosa* (Fig. 4F). Kyoto Encyclopedia of Genes and Genomes (KEGG) analysis and heatmap assessment revealed that the significantly differentially expressed genes (DEGs) were enriched mainly in pathways correlated with LPS biosynthesis, flagellar assembly, histidine metabolism, bacterial RNA transcription, and the regulation of biofilm formation (Fig. 4G to I). The antibacterial mechanism of Cu_x_Te nanozymes against *P. aeruginosa* is illustrated in Fig. 4J. Cu_x_Te nanozymes approach negatively charged bacteria through electrostatic interactions. This subsequently caused initial damage to bacteria via surface spikes. Then, Cu_x_Te nanozymes utilize their multienzyme activity to generate reactive ROS to attack bacteria. Moreover, it down-regulated genes related to LPS and flagellar synthesis, thereby reducing the resistance of gram-negative bacteria. Furthermore, Cu_x_Te nanozymes inhibited bacterial proliferation and division by affecting histidine metabolism and bacterial RNA transcription. Additionally, Cu_x_Te nanozymes suppressed biofilm formation by influencing proteins related to biofilm formation. This multimodal antibacterial mechanism makes gram-negative bacteria vulnerable and unable to escape.

### Specific killing mechanism of the gram-negative bacteria of Cu_x_Te nanozymes

**Fig. 5.**
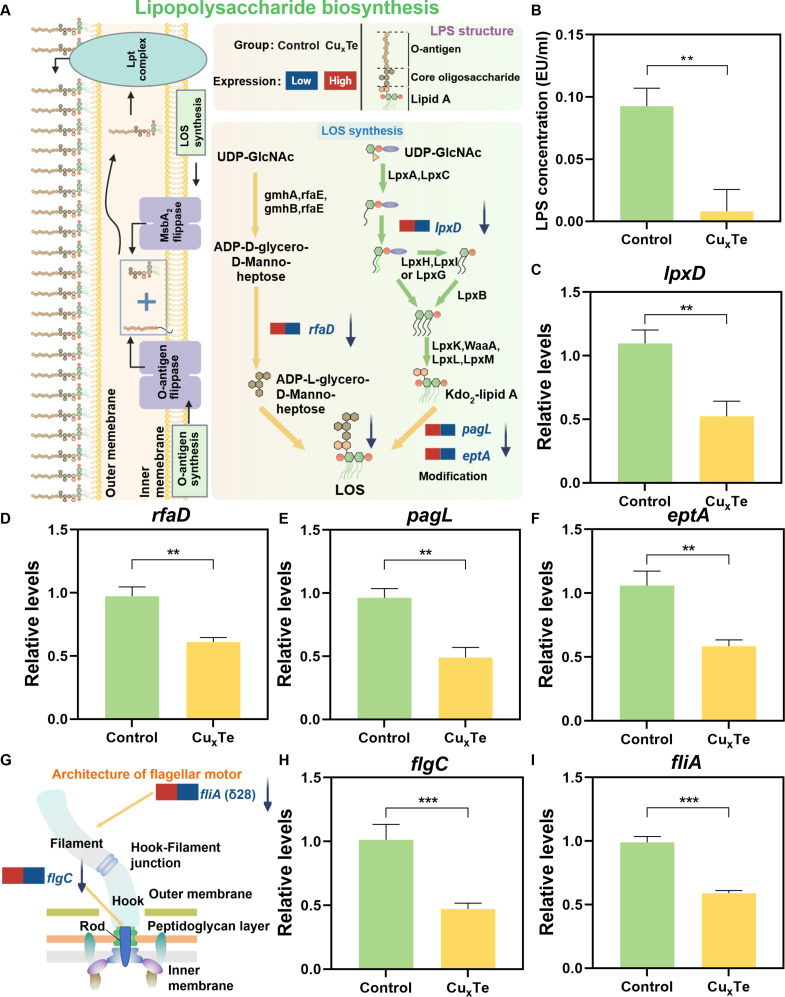
Specific killing mechanism of the gram-negative bacteria of Cu_x_Te nanozymes. (A) Schematic diagram of the mechanism by which Cu_x_Te nanozymes affect lipopolysaccharide biosynthesis in *P. aeruginosa* (red = up-regulation, blue = down-regulation, the left group as Control and the right group as Cu_x_Te) (created in BioRender)*.* (B) LPS concentration in *P. aeruginosa* in Cu_x_Te nanozymes (20 μg/ml) and the control group. (C to F) qPCR results of important genes related to LPS synthesis. (G) Schematic diagram of the mechanism by which Cu_x_Te nanozymes affect the flagellar assembly of *P. aeruginosa.* (H and I) qPCR results of important genes related to flagellar assembly. Statistical analysis was performed using one-way ANOVA followed by Tukey’s post hoc test (**P < 0.01 and ***P < 0.001) (mean ± SD, *n* = 3).

The molecules on the bacterial surface play crucial roles in interactions and reactions with the adjacent environmental context. The exterior layer of gram-negative bacteria consists of an asymmetrical outer membrane, which contains glycolipids known as LPS on its outer layer [28,29]. LPS serves as a fundamental constituent of the outer membrane of most gram-negative bacteria and plays crucial roles in bacterial resistance, virulence, cell division, and the proinflammatory response in the host [2,29,30]. The synthesis of LPS takes place at the cytoplasmic side of the inner membrane and subsequently undergoes translocation across the inner membrane to reach the outer membrane. LPS can be categorized into 3 distinct regions: the invariant lipid A moiety, the central core oligosaccharide segment, and the variable O-antigen portion [30]. LPS synthesis begins with lipooligosaccharide (LOS) synthesis (Fig. 5A), which consists of a sequence of invariant enzymatic reactions that yield the first unit of 3-deoxy-D-manno-octulosonic acid (Kdo) sugar for lipid A and the core oligosaccharide. The lipid A unit is a diphosphorylated disaccharide of glucosamine (GlcN), typically with 4 to 7 acyl chains. The precursors for synthesis are UDP-N-acetylglucosamine (UDP-GlcNAc) and fatty acids bound to acyl carrier proteins. Through the sequential actions of the acyltransferase lpxA, the deacetylase lpxC, and the acyltransferase lpxD, UDP-2,3-diacylglucosamine is subsequently produced. A series of subsequent catalytic processes lead to the formation of Ko2-lipid A. After modification by enzymes such as pagL and eptA, it combines with ADP-L-glycero-D-mannoheptose (core oligosaccharide), which is catalyzed by enzymes such as rfaD, to form LOS [30]. Transcriptome analysis revealed that Cu_x_Te could significantly inhibit the expression of genes such as lpxD, pagL, eptA, and rfaD. These down-regulated genes were further validated via quantitative real-time polymerase chain reaction (qPCR) (Fig. 5C to F). Therefore, combined with the above biosynthetic pathway of LPS, Cu_x_Te nanozymes could effectively inhibit the synthesis of core oligosaccharides and lipid A. To further validate the ability of Cu_x_Te nanozymes to inhibit LPS synthesis, Cu_x_Te nanozymes were incubated with *P. aeruginosa.* After 4 h, an endotoxin detection kit (based on the Limulus amebocyte lysate chromogenic method) was used to test the bacteria in both the control group and the Cu_x_Te group [31]. The endotoxin content in the Cu_x_Te group was significantly lower than that in the control group, further confirming the ability of Cu_x_Te nanozymes to inhibit the LPS synthesis pathway.

Flagella are a fundamental component of most gram-negative bacteria and can increase bacterial pathogenicity through motility and other mechanisms [32,33]. For example, this organelle is associated with bacterial pathogenicity by promoting adhesion [34], invasion [35], chemotaxis [36,37], and bacterial biofilm formation [38]. The bacterial flagellar structure was shown (Fig. 5G). The sequencing results indicate that Cu_x_Te nanozymes can markedly suppress the expression of the *flgC* and *fliA*(δ28) genes, and this result was further validated via qPCR (Fig. 5H and I). The flagellar basal body rod protein is encoded and regulated mainly by the *flgC* gene [39]. Studies have shown that down-regulation of the *flgC* gene leads to a deficiency in *fliA* synthesis and filament assembly, as well as reduced motility and adhesion [40]. Additionally, the protein determined by the *fliA* gene is involved in the formation of flagellar filament subunits, which are assembled into a complete flagellar structure through specific promoters and translational activity [41]. Moreover, the output product of the *fliA* gene is involved in the transcriptional and translational regulation of the flagellum, ensuring its normal function and motility [42]‌. Overall, Cu_x_Te nanozymes could reduce bacterial attachment, invasion, and biofilm formation by inhibiting the expression of the *flgC* and *fliA* genes, thereby suppressing the assembly of bacterial flagella. Therefore, the specific bactericidal effect of Cu_x_Te on gram-negative bacteria was mainly due to the enhanced attack of ROS catalyzed by Cu_x_Te nanozymes, which was amplified by the inhibition of LPS and flagella by Cu_x_Te nanozymes.

### In vitro antibiofilm activity

**Fig. 6.**
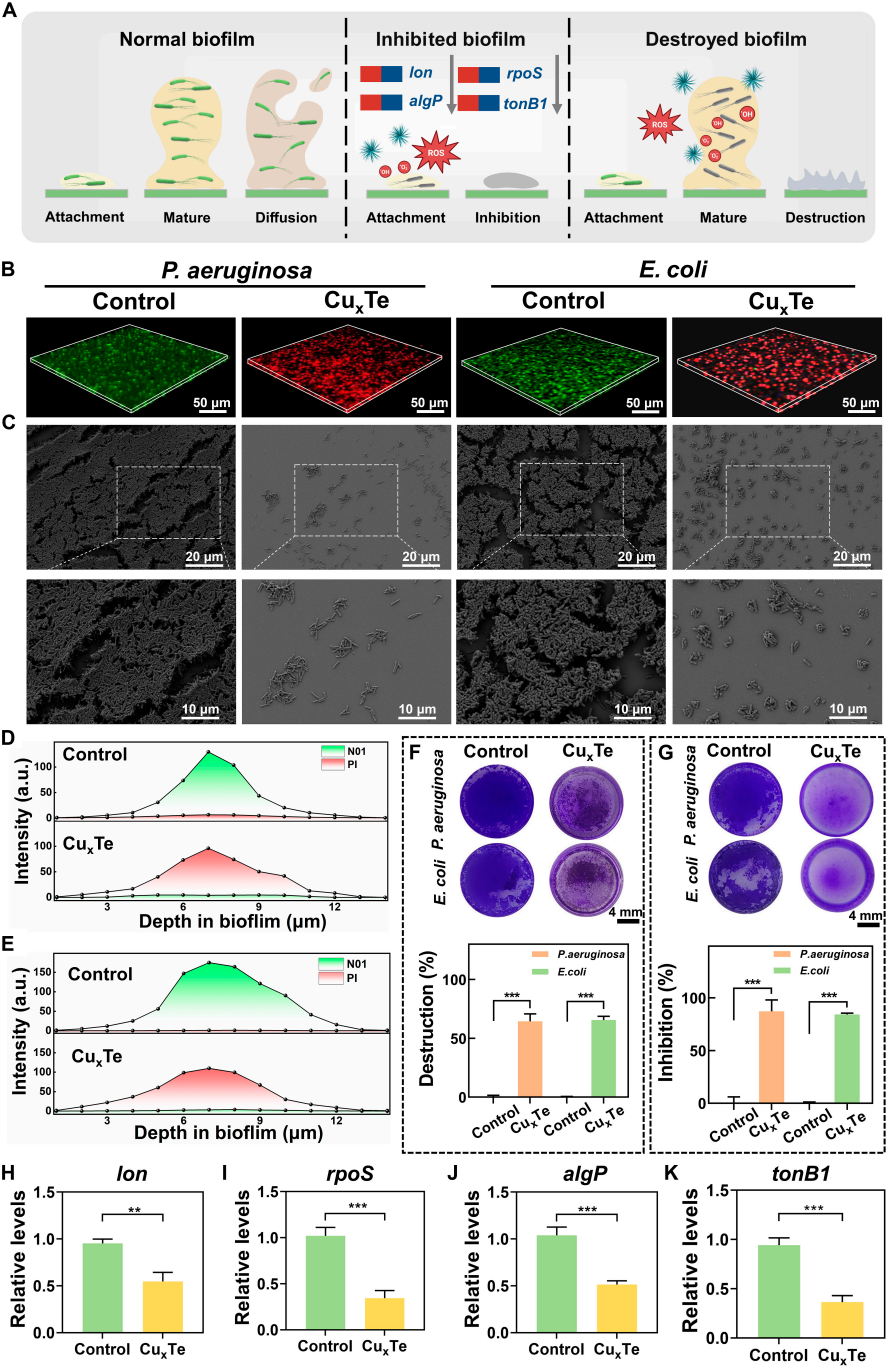
In vitro antibiofilm activity. (A) Schematic diagram of antibiofilm activity in vitro (created in BioRender). (B) Representative images of 3D reconstructions of *P. aeruginosa* and *E. coli* biofilms in the Cu_x_Te (20 μg/ml) and control groups (scale bar, 50 μm). (C) Representative SEM images of *P. aeruginosa* and *E. coli* biofilms in the Cu_x_Te (20 μg/ml) and control groups. (D and E) Quantitative fluorescence statistics of live and dead biofilms in the Cu_x_Te (20 μg/ml) and control groups. (F and G) Photographs and statistical data of *P. aeruginosa* and *E. coli* biofilms stained with crystal violet in the Cu_x_Te (20 μg/ml) and control groups. (H to K) qPCR results of important genes related to biofilm formation. Statistical analysis was performed using one-way ANOVA followed by Tukey’s post hoc test (**P < 0.01 and ***P < 0.001) (mean ± SD, *n* = 3).

Approximately 80% of chronic and refractory infections are intimately associated with biofilm development [43]. Unlike planktonic bacteria, biofilms are equipped with a dense EPS barrier and a BME filled with acidity and H_2_O_2_, which can effectively protect bacteria against host immune defenses and hinder the penetration of antimicrobials, thereby severely impacting the healing of burn wounds [44]. Therefore, the ability of Cu_x_Te nanozymes to combat biofilms is crucial for the treatment of burn wounds. Schematic diagrams of a normal biofilm and a biofilm treated with Cu_x_Te nanozymes are shown in Fig. 6A. A normal biofilm typically undergoes 3 stages: first, the attachment stage, where planktonic bacteria begin to accumulate; subsequently, the biofilm gradually forms and matures; and finally, the mature biofilm enters the diffusion stage, releasing planktonic bacteria to continue “expanding their territory” [45]. When Cu_x_Te nanozymes were introduced during the attachment stage, their unique multimodal antibacterial mechanism markedly affected bacterial survival at this stage and further regulated the expression of genes closely related to biofilm formation, including *lon*, *rpoS*, *algP*, and *tonB1*, thereby effectively inhibiting the formation process of mature biofilms. When Cu_x_Te nanozymes were introduced during the mature stage of the biofilm, they could exert a stronger catalytic effect in a microenvironment filled with acidity and H_2_O_2_ within the biofilm, generating a large amount of ROS to disintegrate and destroy the already formed biofilm structure. The specific experimental data are as follows. Confocal 3D imaging and SEM experiments on biofilms of *P. aeruginosa* and *E. coli* revealed that, in comparison to the control group, the Cu_x_Te nanozymes (30 μg/ml) group had significant bactericidal and destructive effects on biofilms (Fig. 6B and C). Subsequent quantitative analysis of the fluorescence intensity in the confocal images of *P. aeruginosa* (Fig. 6D) and *E. coli* (Fig. 6E) also demonstrated the ability of Cu_x_Te nanozymes to disrupt the biofilms of gram-negative bacteria. The results of the biofilm crystal violet assays for biofilm destruction (Fig. 6F) and biofilm inhibition (Fig. 6G) demonstrated that Cu_x_Te nanozymes not only could destroy biofilms (with disruption rates of 64.46% and 65.61% for *P. aeruginosa* and *E. coli*, respectively) but also effectively inhibited biofilm formation (with inhibition rates of 87.28% and 84.21% for *P. aeruginosa* and *E. coli*, respectively). The results of the transcriptomics study of *P. aeruginosa* revealed that Cu_x_Te nanozymes significantly down-regulated the expression of the biofilm formation genes *lon*, *rpoS*, *algP*, and *tonB1*, and further verification was conducted via qPCR (Fig. 6H to K). The Lon protease is a protein regulated by the *lon* gene. Studies have shown that *lon* deficiency can lead to reduced bacterial adhesion and motility, thereby affecting biofilm formation [46]. *rpoS* is pivotal in regulating the extent of biofilm development, dictating the onset of biofilm initiation, and facilitating the production of extracellular components that substantially enhance biofilm formation [47]. *algP* is essential for the generation of the mucoid exopolysaccharide alginate in *P. aeruginosa*, and mucoid and alginate are important components of biofilm formation in *P. aeruginosa.* Therefore, *algP* deficiency inhibits biofilm formation [8,48]. The *tonB1* protein family affects iron transport in gram-negative bacteria, thereby influencing biofilm formation [49]. As a result, the excellent antibiofilm ability of Cu_x_Te nanozymes can be ascribed to 2 aspects: on the one hand, they can catalyze a large amount of ROS in the BME; on the other hand, they can inhibit the manifestation of genes associated with biofilm development.

### Performance characterization and antibacterial activity of the Cu_x_Te@CG hydrogel

**Fig. 7.**
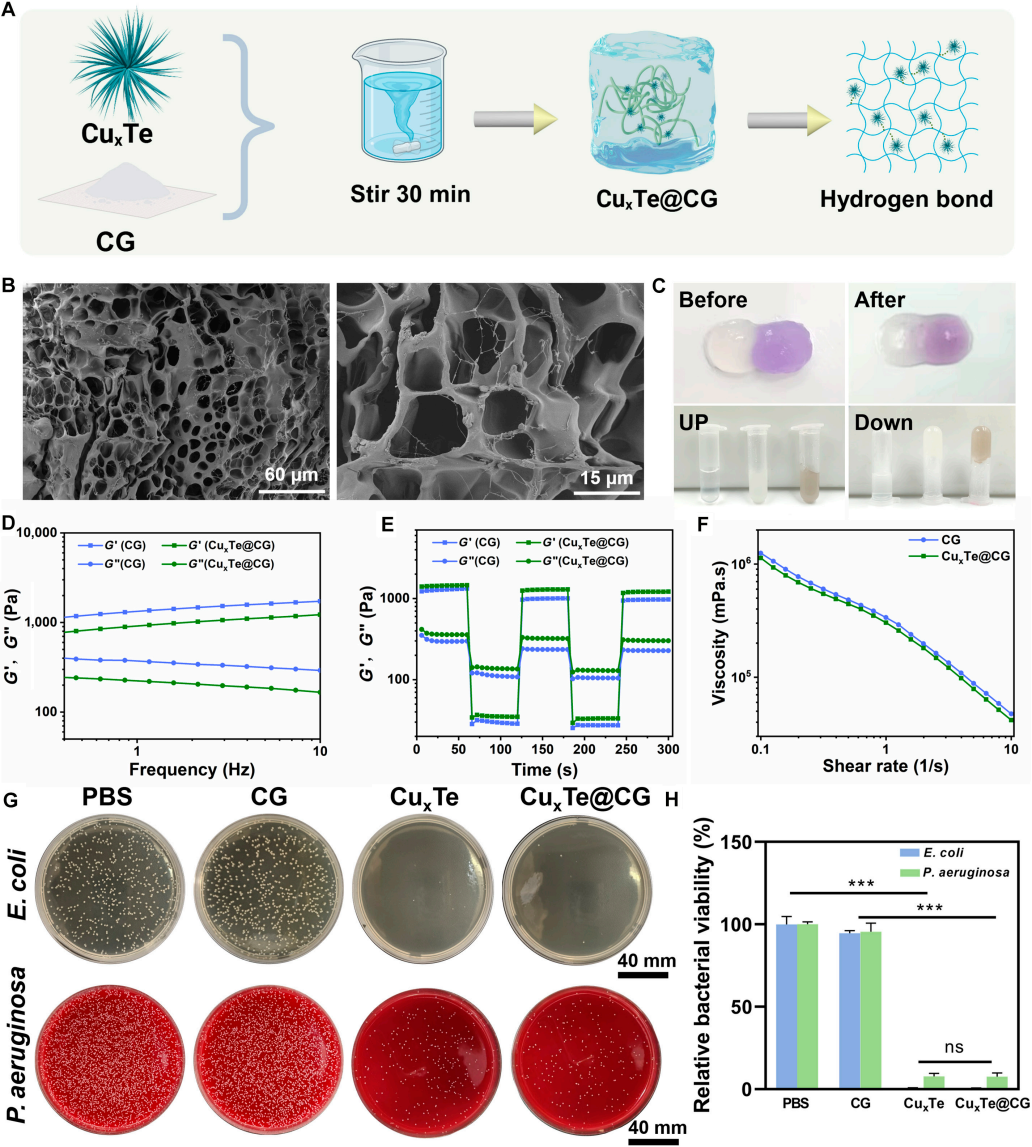
Performance characterization and antibacterial activity of the Cu_x_Te@CG hydrogel. (A) Schematic diagram of Cu_x_Te@CG hydrogel synthesis (created in BioRender). (B) SEM images of the Cu_x_Te@CG hydrogel (with a Cu_x_Te concentration of 20 μg/ml). (C) Self-healing and adhesive properties of the Cu_x_Te@CG hydrogel (with a Cu_x_Te concentration of 20 μg/ml). (D to F) Rheological properties of the Cu_x_Te@CG hydrogel (with a Cu_x_Te concentration of 20 μg/ml): frequency sweep, dynamic step strain, and viscosity measurements. (G) Photographs of *P. aeruginosa* and *E. coli* bacterial colonies cocultured with different groups (PBS, CG, Cu_x_Te, and Cu_x_Te@CG [with a Cu_x_Te concentration of 20 μg/ml]). (H) Relative bacterial viability of *P. aeruginosa* and *E. coli* bacteria after different treatments. Statistical analysis was performed using one-way ANOVA followed by Tukey’s post hoc test (***P < 0.001) (mean ± SD, *n* = 3).

To expand the clinical application scenarios of Cu_x_Te nanozymes with excellent antibacterial activity, we blended them with hydrogels, an ideal wound dressing, for the treatment of mice [50]. By uniformly mixing the previously described urchin-like nanozyme Cu_x_Te with cationic guar gum (CG), Cu_x_Te nanozymes could be successfully loaded into CG, changing its color from the original milky white to light gray, thus forming the Cu_x_Te@CG nanozyme hydrogels (Fig. 7A). The SEM image of the Cu_x_Te@CG hydrogel revealed the microstructure and morphology of the hydrogel (Fig. 7B). The SEM image revealed that the hydrogel had a loose and porous structure, which could easily accommodate Cu_x_Te nanozymes (with a particle size of approximately 200 nm). Upon further observation, many small and uniformly sized particles were encapsulated within the hydrogel matrix. The aforementioned findings confirmed the successful preparation of the Cu_x_Te@CG hydrogel. The excellent self-healing and adhesive properties of the Cu_x_Te@CG hydrogel were proven (Fig. 7C). When a regular Cu_x_Te@CG hydrogel sample was brought into close contact with a Cu_x_Te@CG hydrogel sample dyed purple, the color boundary between the 2 became blurred over time, and the gap at the junction became smoother. This indicated the good self-healing property of the Cu_x_Te@CG hydrogel. Additionally, when PBS, CG, and the Cu_x_Te@CG hydrogel were added to centrifuge tubes and inverted for 30 min, neither CG nor the Cu_x_Te@CG hydrogel flowed back, confirming the strong adhesive capabilities of the Cu_x_Te@CG hydrogel. The experiment indirectly revealed that the Cu_x_Te@CG hydrogel adhered well to and adapted to irregular burn wounds (Fig. 7C). When the Cu_x_Te@CG hydrogel was injected into the bottom of a Petri dish, the “AHMU” letters remained stable in the hydrogel state, demonstrating the injectability and stability of the hydrogel (Fig. S6). Next, in this study, the mechanical characteristics of the CG and Cu_x_Te@CG hydrogel were systematically evaluated. In the dynamic frequency scan experiment, as the frequency gradually increased from 0.1 to 10 Hz, the storage modulus (*G′*) consistently remained greater than the loss modulus (*G*″), indicating that the Cu_x_Te@CG hydrogel possesses superior elastic properties. A dynamic step-strain rheological experiment was subsequently performed to quantitatively confirm the self-recovery capability of the designed hydrogel (Fig. 7D). Initially, the changes in *G′* and *G*″ of the Cu_x_Te@CG hydrogel were measured through rheological recovery tests. A minor strain of 1% alongside a substantial strain of 400% (more than 4 times the critical point) was chosen. When the dynamic strain was adjusted to a relatively high value (400%), *G′* decreased below *G*″, confirming that the hydrogel underwent significant deformation. However, once the applied dynamic strain was restored to 1%, *G′* sharply increased and instantly surpassed *G*″, suggesting that the specimen had reverted to its hydrogel state (Fig. 7E). Furthermore, both *G′* and *G*″ fully recovered to their original values. Notably, even after 3 cycles, this phase transition remained consistent, demonstrating the remarkable deformation recovery capability of the Cu_x_Te@CG hydrogel (Fig. 7E). Additionally, the viscosity curve of this biomedical hydrogel was further investigated. With the escalation of the shear rate, the viscosity of the hydrogel progressively diminished, demonstrating its injectability (Fig. 7F). Subsequently, strain experiments demonstrated that the probability of intersection between the CG hydrogel and the Cu_x_Te@CG hydrogel was 100% (Fig. S11).

The antibacterial efficacy of the Cu_x_Te@CG hydrogel against *P. aeruginosa* and *E. coli* was evaluated via the dilution spread plate method. The results revealed no significant difference between the control group and the CG group, indicating that CG itself does not possess antibacterial activity. Compared with the PBS and CG groups, both the Cu_x_Te and Cu_x_Te@CG groups exhibited statistically significant differences in bacterial viability, suggesting that both the Cu_x_Te and Cu_x_Te@CG groups exhibit excellent antibacterial activity. Additionally, there was no significant difference between the Cu_x_Te and Cu_x_Te@CG groups, indicating that the encapsulation of Cu_x_Te nanozymes by CG did not affect its antibacterial performance. The bacterial survival rates of *E. coli* and *P. aeruginosa* decreased to below 10% (Fig. 7G and H). These results confirmed that the Cu_x_Te@CG hydrogel possessed outstanding properties, including excellent antibacterial ability, self-healing capacity, injectability, stability, and adhesion, making it a potential therapeutic agent for effectively eliminating bacterial infections associated with burn wounds and filling damaged burn wound tissues.

### In vivo therapeutic effect of the Cu_x_Te@CG hydrogel on *P. aeruginosa*-infected burn wounds

**Fig. 8.**
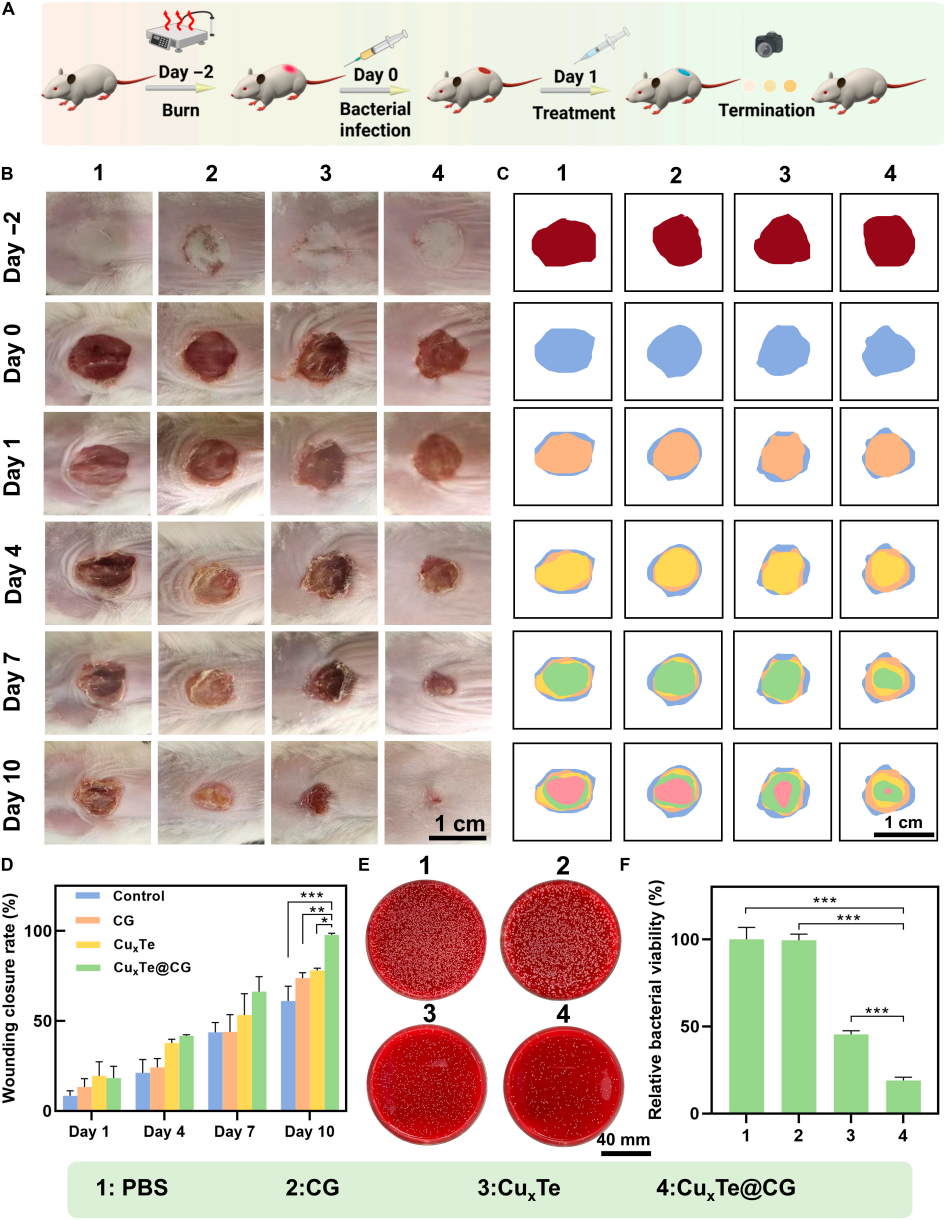
In vivo therapeutic effect of the Cu_x_Te@CG hydrogel on *P. aeruginosa*-infected burn wounds. (A) Schematic diagram of the burn wound infection model establishment and therapeutic pathway (created in BioRender). (B) Photographs of burn wounds and (D) percentage of wounds on mice after various treatments for 10 days in different treatment groups. (C) Wound healing trajectory depicted. (E) Photographs and (F) quantitative analysis of bacterial colonies obtained from wound tissues after various treatments. Statistical analysis was performed using one-way ANOVA followed by Tukey’s post hoc test (*P < 0.05, **P < 0.01, and ***P < 0.001) (mean ± SD, *n* = 5).

The excellent antibacterial and antibiofilm capabilities of the Cu_x_Te nanozymes were verified through in vitro experiments. In subsequent experiments, a *P. aeruginosa*-infected burn wound model in mice was established to investigate the in vivo antibacterial efficacy and therapeutic potential of the Cu_x_Te@CG hydrogel. A schematic diagram (Fig. 8A) illustrates the establishment of the *P. aeruginosa*-infected burn wound model and the subsequent treatment process with the Cu_x_Te@CG hydrogel. On day −2, the burn model was established, followed by debridement on day 0, and then *P. aeruginosa* was injected into the wound to induce infection for 24 h. The mice were subsequently randomly allocated into 4 groups: the PBS, CG, Cu_x_Te, and Cu_x_Te@CG groups. Images of the mouse wounds were captured, and wound healing traces were documented (Fig. 8B and C) over the 10 days following different treatments. The results indicated that the Cu_x_Te@CG hydrogel treatment had the best therapeutic effect, with nearly complete wound healing by the 10th day. The average wound healing rates for the PBS, CG, Cu_x_Te, and Cu_x_Te@CG groups on the 10th day were 61.06%, 73.78%, 78.09%, and 97.76%, respectively (Fig. 8D). These findings demonstrate the excellent therapeutic efficacy of the Cu_x_Te@CG hydrogel for burn-infected wounds. On the tenth day, the wound tissues and exudates were collected, and bacterial cultures were performed to measure the residual bacterial load in the burn tissues after treatment. The results revealed that the Cu_x_Te@CG group had the lowest bacterial count on the 10th day (Fig. 8E and F). Compared with those in the PBS and CG groups, the significant difference in bacterial count observed in the Cu_x_Te@CG group was attributed primarily to the superior antibacterial properties of Cu_x_Te nanozymes. Furthermore, compared with the Cu_x_Te group, the enhanced antibacterial effect was due to the outstanding characteristics of the CG hydrogel: after loading with Cu_x_Te nanozymes, the Cu_x_Te@CG hydrogel could tightly adhere to the wound site, enabling stable and long-lasting antibacterial action. Therefore, these results indicate that the Cu_x_Te@CG hydrogel has excellent anti-infective capabilities and promotes burn wound healing.

### Slice staining and immunofluorescence staining images of the wound tissues

**Fig. 9.**
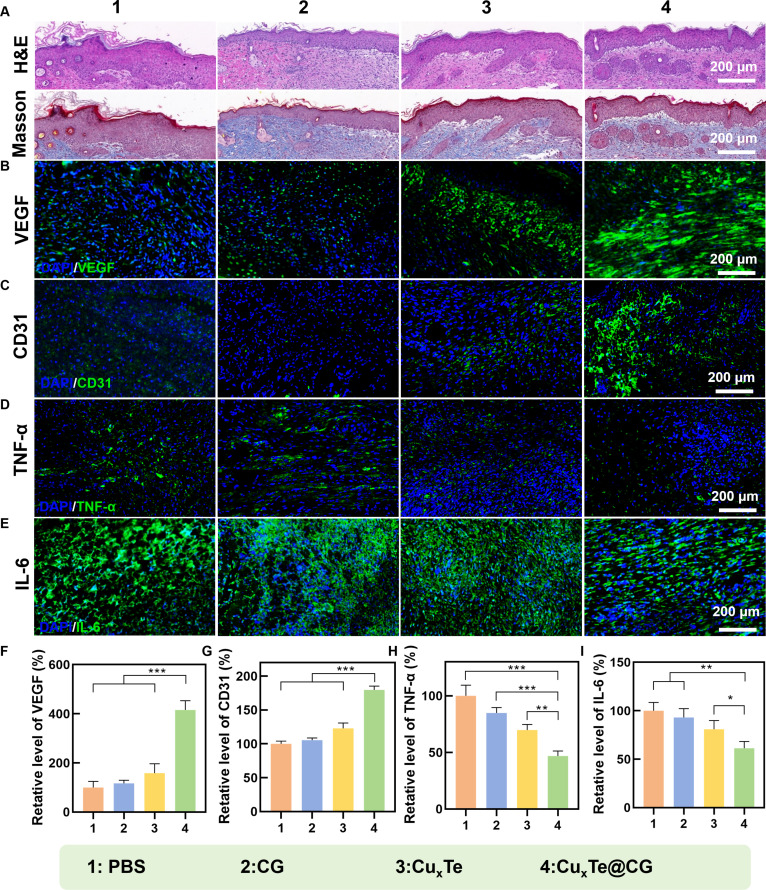
Slice staining and immunofluorescence staining images of the wound tissues. (A) H&E and Masson’s trichrome staining of wounds from different treatment groups on day 10. (B to E) Immunofluorescence images of VEGF, CD31, TNF-α, and IL-6 expression on day 10. (F to I) Statistical analysis of the VEGF, CD31, TNF-α, and IL-6 levels in the wounds. Statistical analysis was performed using one-way ANOVA followed by Tukey’s post hoc test (*P < 0.05, **P < 0.01, and ***P < 0.001) (mean ± SD, *n* = 5).

On the basis of the experimental results from the previous section, the Cu_x_Te@CG hydrogel had an exceptionally outstanding ability to promote healing in burn wounds infected with *P. aeruginosa.* Therefore, the subsequent study preliminarily explored the mechanism by which the Cu_x_Te@CG hydrogel promoted healing through slice staining and immunofluorescence staining images of wound tissues. The results of hematoxylin and eosin (H&E) staining and Masson’s trichrome staining indicated that, compared with those in the other groups, the tissues in the Cu_x_Te@CG group presented more intact tissue structures and organ morphologies (Fig. 9A). During the healing of burn wounds, certain factors that promote cell growth regulation and inflammatory responses play crucial roles. The immunofluorescence results demonstrated that the tissue samples treated with the Cu_x_Te@CG hydrogel had significantly higher levels of vascular endothelial growth factor (VEGF) and endothelial cell adhesion molecule (CD31) than the other treatment groups (Fig. 9B, C, F, and G), which was likely attributed to the slow release of copper ions during the treatment process with the Cu_x_Te@CG hydrogel. Moreover, the tissue samples from the mice treated with the Cu_x_Te@CG hydrogel presented significantly lower levels of the proinflammatory cytokines TNF-α and IL-6 than those from the other groups (Fig. 9D, E, H, and I). This was likely due to the antibacterial activity of the Cu_x_Te@CG hydrogel, which inhibits the inflammatory response at the burn wound site. Additionally, Cu_x_Te nanozymes inhibited the synthesis pathway of LPS, which could also contribute to the low levels of inflammatory cytokines. The above results indicated that the Cu_x_Te@CG hydrogel promoted blood vessel formation through the action of copper ions and reduced the inflammatory response at the burn tissue site through its excellent antibacterial activity and inhibition of LPS.

### RNA sequencing analysis of the therapeutic effect of the Cu_x_Te@CG hydrogel

**Fig. 10.**
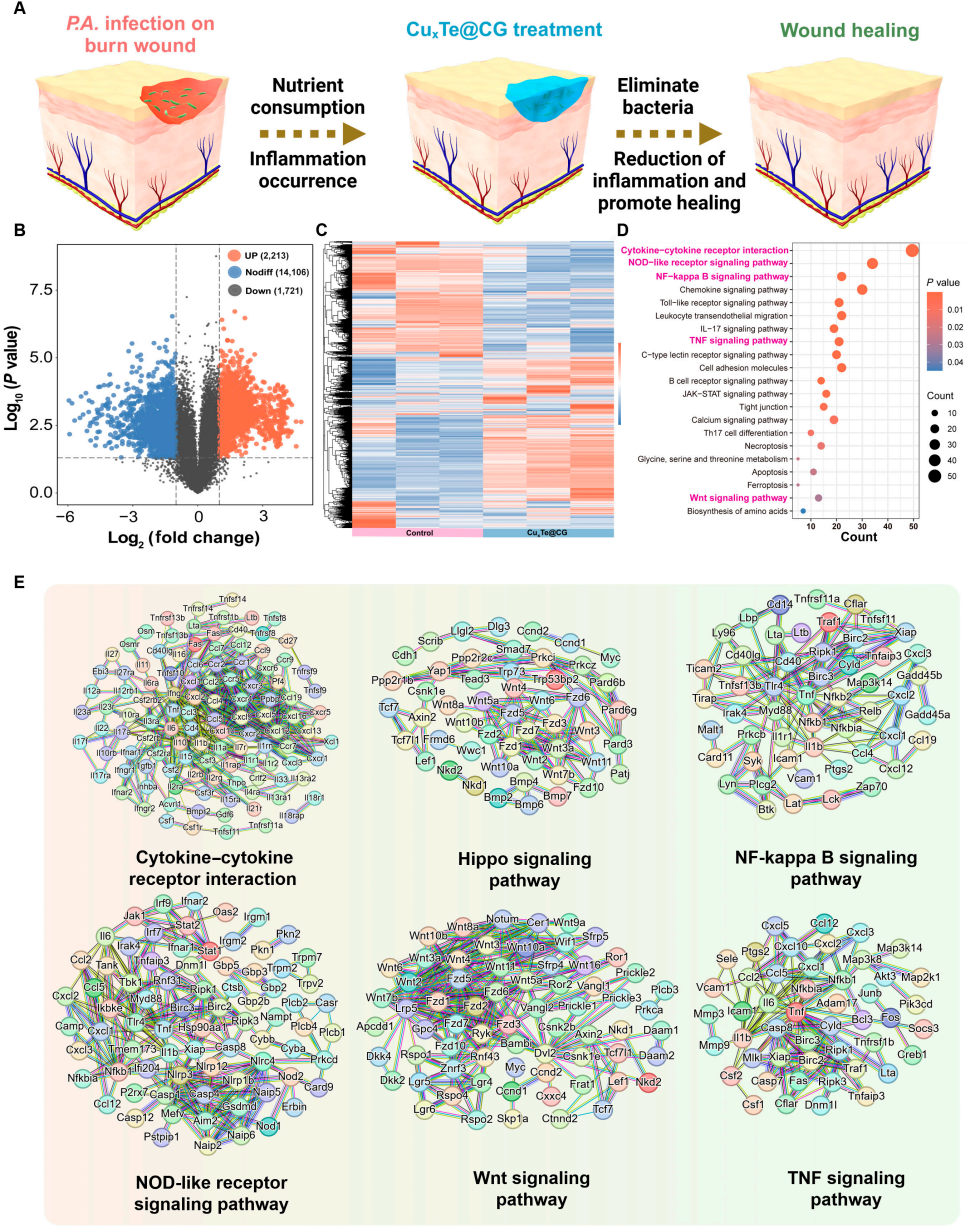
RNA sequencing analysis of the therapeutic effect of the Cu_x_Te@CG hydrogel. (A) Schematic illustration of Cu_x_Te@CG hydrogel-enhanced healing mechanisms in *P. aeruginosa-*infected burn wounds. (B) Volcano map of up-regulated (red) and down-regulated (blue) genes. (C) Heatmap of the expression of genes. (D) KEGG pathway enrichment analysis. (E) Protein–protein interaction (PPI) network diagram.

In the previous sections, the therapeutic effect and healing promotion mechanism of the Cu_x_Te@CG hydrogel were demonstrated. After a burn wound is infected with *P. aeruginosa*, the proliferation of bacteria greatly consumes nutrients at the wound site and is accompanied by an inflammatory response, which makes healing of infected burn wounds very difficult (Fig. 10A). After treatment with the Cu_x_Te@CG hydrogel, many bacteria were eliminated, effectively controlling infection and inflammation at the burn wound site and ultimately promoting wound healing. To further elucidate the molecular mechanisms underlying the promotion of wound healing by the Cu_x_Te@CG hydrogel, high-throughput RNA sequencing (RNA-seq) was employed to investigate the differential gene expression profiles between skin tissues treated with the Cu_x_Te@CG hydrogel and those in the control group. A total of 3,934 DEGs were identified (log2-fold change > 1.00; *P*_adj_ < 0.05). A volcano plot was constructed, which revealed 2,213 up-regulated genes and 1,721 down-regulated genes after Cu_x_Te@CG hydrogel treatment (Fig. 10B). A heatmap of the up-regulated and down-regulated DEGs is shown (Fig. 10C). Gene Ontology (GO) enrichment analysis indicated that the skin formation, cell differentiation, signal transduction, chemotaxis, and inflammatory response of the tissues treated with the Cu_x_Te@CG hydrogel were significantly affected (Fig. S12). KEGG pathway enrichment analysis revealed that the markedly DEGs were primarily clustered in critical regulatory networks, including the cytokine–cytokine receptor interaction cascade, NOD-like receptor-mediated innate immune signaling, NF-kappa B-driven inflammatory modulation, TNF-α-associated tissue repair pathways, and Wnt-dependent regenerative signaling. Collectively, the coordinated activation of these pathways likely accelerates burn wound healing by orchestrating inflammation resolution, stimulating keratinocyte migration, and promoting extracellular matrix remodeling (Fig. 10D). Furthermore, protein–protein interaction network analysis elucidated the intricate crosstalk and functional associations among proteins within the aforementioned DEG-enriched pathways, thereby providing mechanistic insights into how Cu_x_Te@CG hydrogel treatment enhances burn wound healing (Fig. 10E). Overall, treatment with the Cu_x_Te@CG hydrogel promoted cell proliferation and differentiation while indirectly reducing inflammation, ultimately achieving excellent therapeutic effects.

### Assessment of biosafety and biocompatibility

**Fig. 11.**
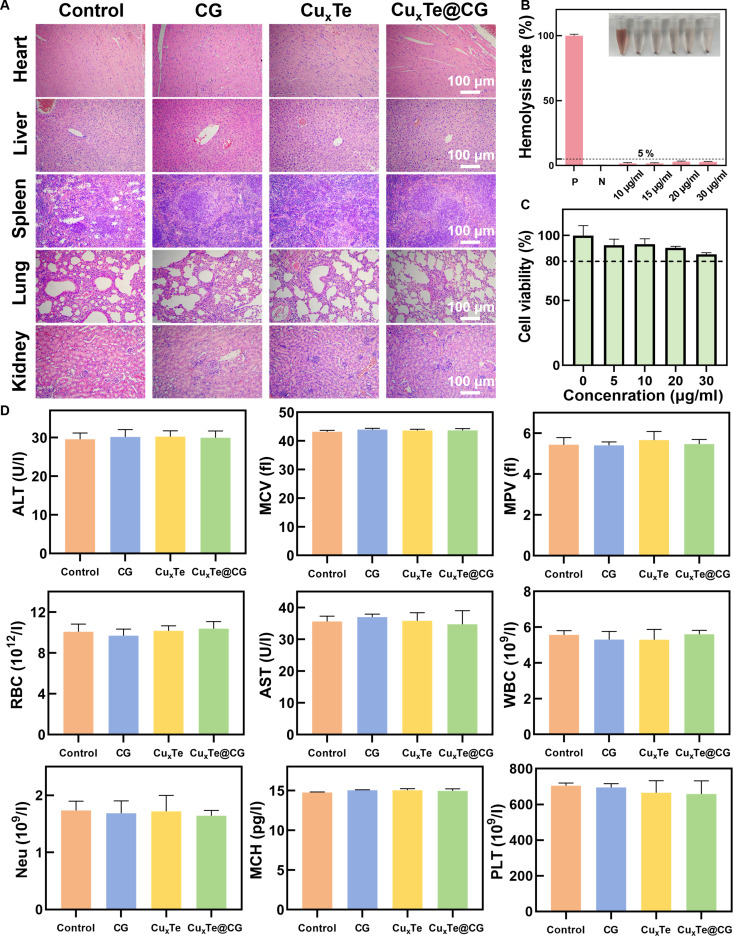
Assessment of biosafety and biocompatibility. (A) H&E sections of major organs (heart, liver, spleen, lung, and kidney) of mice in different groups on day 10. (B) Hemolysis rates and digital photographs of red blood cells after different treatments. (C) Cytotoxicity of the Cu_x_Te@CG hydrogel to L929 cells evaluated by a CCK-8 assay. (D) Routine blood and biochemical parameters of normal mice and other treatment groups on day 10. Statistical analysis was performed using one-way ANOVA followed by Tukey’s post hoc test (mean ± SD, *n* = 5).

In vitro experiments using mouse fibroblasts (L929) were conducted to evaluate the biocompatibility of the Cu_x_Te@CG hydrogel for biomedical applications. Even at a concentration of 30 μg/ml of Cu_x_Te nanozymes in the Cu_x_Te@CG hydrogel, 85.5% cell viability was observed, complying with biocompatibility standards (Fig. 11C). Furthermore, the results of the hemolysis test demonstrated the excellent blood compatibility of the Cu_x_Te@CG hydrogel (Fig. 11B). In vivo, histological analysis of heart, liver, spleen, lung, and kidney samples from the 4 groups on day 10 revealed no apparent tissue abnormalities (Fig. 11A). Additionally, there were no significant differences in physiological blood parameters among the groups, indicating the superior biocompatibility of the Cu_x_Te@CG hydrogel (Fig. 11D). These results collectively demonstrated that the Cu_x_Te@CG hydrogel could serve as a safe and low-toxicity antibacterial treatment for burn wounds.

## Conclusion

In summary, this work presents a Cu_x_Te@CG hydrogel that acts as a gram-negative specific bactericidal platform specific for the treatment of burn wounds infected by *P. aeruginosa*. This platform exerts synergistic oxidase- and glutathione peroxidase-mimetic effects. By catalyzing ROS generation and depleting bacterial GSH, it efficiently triggers lethal oxidative stress to eliminate bacteria. Bacterial transcriptomic data demonstrated that the Cu_x_Te@CG hydrogel interferes with the synthesis processes of LPS and flagella unique to gram-negative bacteria, thereby further amplifying and enhancing the attack and destructive effects of ROS on bacteria. Animal experiments demonstrated that treatment with the Cu_x_Te@CG hydrogel effectively inhibited *P. aeruginosa* infection in burn wounds. Furthermore, in tissue samples treated with the Cu_x_Te@CG hydrogel, factors promoting angiogenesis were significantly up-regulated, whereas proinflammatory factors were significantly down-regulated, as confirmed by the experimental and transcriptome sequencing results. Overall, the Cu_x_Te@CG hydrogel prepared in this study serves as a novel strategy for treating *P. aeruginosa*-induced burn wounds while also offering new insights into combating highly drug-resistant gram-negative bacteria.

## Materials and Methods

### Materials

Anhydrous copper chloride (CuCl_2_), telluric acid (H_6_TeO_6_), ascorbic acid (C_6_H_8_O_6_, AA), and benzyl alcohol (C_7_H_8_O, BP) were purchased from Aladdin, and PVP (molecular weight 40,000) was purchased from Macklin. OPD, DTNB, and GSH were purchased from Aladdin, and an LPS detection kit, tryptone soybean broth, agar powder (C_12_H_18_O_9_)*_n_*, and a bacterial life and death staining kit (500 T) were purchased from BestBio. DMPO was purchased from Dojindo Laboratories (Japan). The MDA Content Detection Kit was purchased from AIDISHENG (ADS-W-YH002). A ROS assay kit was purchased from Beyotime Company (China). Deionized water was prepared via a purification system (Direct-Q3, Millipore, USA). A chromogenic LAL endotoxin assay kit (160T) was purchased from Beyotime (China).

### Preparation of the Cu_x_Te nanozymes

A total of 20 ml of benzyl alcohol (BP) solution was added, and the mixture was poured into a cleaned flask for later use. One hundred milligrams of PVP, 36 mg of ascorbic acid (AA), 8.2 mg of CuCl_2_, and 11.6 mg of H_6_TeO_6_ were weighed and poured sequentially into a flask containing BP. The mixed flask was subsequently placed in an ultrasonic cleaner for ultrasonic treatment to thoroughly mix the solutes. Next, the mixture was heated to 160 °C and incubated in an oil bath for 3 h. After the heating process, the mixture was allowed to cool naturally. The prepared solution was centrifuged to obtain a black–brown precipitate. The black–brown product was subsequently washed 3 times with ethanol, acetone, or water. Finally, the mixture was placed in a freeze dryer for freeze-drying to obtain Cu_x_Te nanozymes.

### Materials characterization

The morphology of the samples was analyzed via field emission SEM (Hitachi S-4800, Japan) and TEM (JEM-1400 Plus, Japan). XRD patterns were measured with an x-ray diffractometer (DX-2700BH, China) at 40 kV and 30 mA. To determine the chemical composition of the samples (Thermo Scientific K-Alpha, China), XPS was employed. An ultraviolet–visible (UV–Vis) spectrophotometer (Thermo Scientific Genesys 50, China) was used to measure the UV–Vis absorption spectrum of the sample. Microplate spectrophotometers (Synergy2 SLFPTAD, USA) were used to measure the optical density (OD).

### OXD-like activity

An OPD probe and DMPO as trapping agents were used to validate the activity of the OXD-like enzymes. The prepared pH 5.5 Cu_x_Te solution (in PBS) was mixed uniformly and then added to OPD (100 μg/ml) probe solutions to create Cu_x_Te concentrations of 0, 10, 20, 30, 40, and 50 μg/ml. The mixtures were then incubated in an oven at 37 °C for 15 min, after which the color changes of the solutions were recorded via a digital camera. The absorbance of the OPD solutions treated with different concentrations of Cu_x_Te nanozymes was subsequently measured at λ = 442 nm via a UV–Vis spectrophotometer (Thermo Scientific Biomate 160 [USA]), and the results were recorded. A 50 μg/ml Cu_x_Te solution in PBS was prepared and added to a DMPO solution dissolved in methanol to test superoxide anions via ESR spectroscopy (Bruker EMXPLUS, Germany).

### GSH oxidase-like activity and peroxidase-like activity

The DTNB probe was used to validate the GSH-Ox-like activity. The prepared Cu_x_Te solution was added to a premade 1.0 mM GSH solution to achieve a Cu_x_Te concentration of 50 μg/ml in the reaction system. The DTNB probe was then added at 0 min, 5 min, 10 min, 20 min, 30 min, 1 h, 1.5 h, 2 h, 3 h, 4 h, and 5 h. The absorbance was measured via a UV–Vis spectrophotometer (Thermo Scientific Biomate 160 [USA]), and the results were recorded. Finally, Origin graphing software was used to plot the curves.

For the GSH oxidase-like activity assay, 4 groups of reaction systems were prepared with a constant Cu_x_Te concentration of 50 μg/ml and the same GSH concentration, including a no gas bubbling group, a nitrogen bubbling group, an air bubbling group, and an oxygen bubbling group; after incubation for 10 min, the GSH concentration in each group was determined using the DTNB probe. For the GSH peroxidase-like activity-related assay, another set of reaction systems was prepared under the conditions of 50 μg/ml Cu_x_Te nanozymes and the same GSH concentration, with exogenous hydrogen peroxide (H₂O₂) added at different concentrations (0, 0.25, 0.5, and 1 mM); the GSH concentration in each group was detected using the DTNB probe after 10 min of incubation.

### Evaluation of the aqueous stability of Cu_x_Te nanozymes

To evaluate the aqueous stability of Cu_x_Te nanozymes, freshly prepared Cu_x_Te nanozymes were dispersed in aqueous solution; on day 1, a portion of the dispersion was sampled for morphology observation and recording via TEM (JEM-1400 Plus, Japan). Subsequently, the oxidase-like activity of Cu_x_Te nanozymes at different concentrations (0, 10, 20, 30, and 40 μg/ml) was determined using the OPD probe under the conditions of pH 5.5, 100 μg/ml OPD, and incubation at 37 °C for 10 min, with the absorbance measured at λ = 442 nm via a UV–Vis spectrophotometer (Thermo Scientific Biomate 160 [USA]). Additionally, the GSH oxidase-like activity was assessed by detecting the content of 1.0 mM GSH (at 0 min, 10 min, 30 min, 1 h, and 2 h of reaction) after oxidation by 50 μg/ml Cu_x_Te nanozymes using the DTNB probe, with detection performed via the same UV–Vis spectrophotometer. All the above-mentioned experiments were repeated on day 10. The copper ion release assay was conducted as follows: freshly prepared Cu_x_Te nanozymes were formulated into a 1 mg/ml aqueous solution and uniformly dispersed via ultrasonication for 5 min. Then, 1 ml of the Cu_x_Te nanozymes aqueous solution was transferred into a dialysis bag, which was subsequently immersed in PBS buffers with pH 7.2, 6.0, and 5.0. The systems were incubated in a constant temperature shaker at 37 °C. At predetermined time points (0 h, 30 min, 1 h, 2 h, 4 h, 8 h, 16 h, 24 h, 48 h, and 96 h), aliquots of the immersion solution were collected, and the copper ion concentration was determined using an inductively coupled plasma optical emission spectrometer (ICP–OES, EXPEC 6500).

### In vitro antibacterial efficiency

In this study, MRSA, *S. aureus*, and *E. faecalis* were used as models for gram-positive bacteria, whereas *E. coli*, *P. aeruginosa*, and *K. pneumoniae* were used as models for gram-negative bacteria. First, the in vitro antibacterial activity of Cu_x_Te nanozymes was evaluated via the spread plate method. Cu_x_Te solutions of different concentrations were incubated with bacteria (MRSA, *E. coli*, and *P. aeruginosa*) at a concentration of 1 × 10^6^ (CFU)/ml. The samples from each group were subsequently added to individual wells of a 96-well plate and incubated statically in a 37 °C incubator for 4 h. The bacteria were then spread onto agar plates, and photographs were taken after 12 h to record and determine the number of colonies via ImageJ software. Next, the treated bacteria from each group were stained with the N01/PI live/dead dye to assess bacterial viability via confocal laser scanning microscopy. To further validate the targeted killing effect of Cu_x_Te nanozymes on gram-negative bacteria, bacterial growth curves were generated for both gram-positive and gram-negative bacteria. Bacterial suspensions (MRSA, *S. aureus*, *E. faecalis*, *E. coli*, *P. aeruginosa*, and *K. pneumoniae*) were added to a 96-well plate, followed by the addition of Cu_x_Te nanozymes to achieve concentrations of 0, 5, 10, and 20 μg/ml. The absorbance of the bacterial suspensions was then measured at different time points. To explore the mechanism of the targeted killing effect of Cu_x_Te nanozymes on gram-negative bacteria, the treated bacteria from each group were stained with a DCFH-DA probe to detect the production of ROS via confocal laser scanning microscopy and flow cytometry. Simultaneously, the samples from different treatments were fixed with 2.5% glutaraldehyde, gradient dehydrated with different concentrations of ethanol, and finally freeze-dried. The bacterial morphology of each group was then observed by SEM on silicon wafers. The resuscitated MRSA, *E. coli*, *P. aeruginosa*, and *K. pneumoniae* were adjusted to an OD value of 0.65 for later use. Sterile 5-ml EP tubes were prepared, and 200 μl of bacteria was added to each tube separately. Subsequently, PBS (pH 5.5) was added to establish 2 groups: a Cu_x_Te-free control group and a Cu_x_Te-treated group with a final Cu_x_Te concentration of 20 μg/ml. All prepared systems were incubated in a 37 °C shaker for 4 h. After incubation, bacteria in each group were collected by centrifugation at 5,000 rpm for 5 min. A total of 5 million bacteria were resuspended in 1 ml of MDA extraction solution, followed by homogenization under ice-bath conditions. The homogenate was centrifuged at 12,000 rpm for 10 min at 4 °C, and the supernatant was collected as the sample to be tested. Then, 200 μl of the sample was mixed with 300 μl of working solution, heated in a 90 to 95 °C water bath for 30 min, and cooled to room temperature. After centrifugation at 12,000 rpm for 10 min at 25 °C, 200 μl of the supernatant was added to a 96-well plate. The absorbance at OD_532_ and OD_600_ was measured using a microplate reader (SpectraMax iD3).

### Preparation, characterization, and antibacterial activity of the Cu_x_Te@CG hydrogel

First, 10 ml of a Cu_x_Te solution at a concentration of 20 μg/ml was prepared, and then 300 mg of CG was added to the Cu_x_Te solution. After rapid stirring for 30 min, the Cu_x_Te@CG hydrogel was obtained. The prepared Cu_x_Te@CG hydrogel was freeze-dried and placed on a silicon wafer for observation of its microscopic morphology via a scanning electron microscope. To assess the self-healing property of the Cu_x_Te@CG hydrogel, part of the Cu_x_Te@CG hydrogel was stained purple with a dye. The stained Cu_x_Te@CG hydrogel was subsequently brought into contact with the unstained Cu_x_Te@CG hydrogel, which was recorded via a digital camera. The process was repeated after 6 h. To further evaluate their self-healing properties, water, CG, and the Cu_x_Te@CG hydrogel were placed in centrifuge tubes and photographed. After the tubes were inverted for 30 min, they were photographed again. A rheometer (Anton Paar MCR 302e [Eur]) was subsequently used to test the rheological properties of CG and the Cu_x_Te@CG hydrogel. To assess the antibacterial activity of the Cu_x_Te@CG hydrogel, 4 groups, PBS, CG, Cu_x_Te, and Cu_x_Te@CG (with a Cu_x_Te concentration of 20 μg/ml), were incubated with *E. coli* and *P. aeruginosa.* After 4 h of incubation, the samples were diluted and spread on plates. Following 12 h of cultivation in an oven, the plates were photographed, and the number of colonies was determined via ImageJ software.

### In vitro antibiofilm properties

For biofilm cultivation, suspensions of cultivated *E. coli* and *P. aeruginosa* (1 × 10^8^ CFU/ml) were added to 900 μl of Luria–Bertani (LB) medium and incubated statically at 37 °C for 48 h to form mature biofilms. First, mature biofilms were incubated on confocal dishes and treated with PBS or Cu_x_Te nanozymes for 12 h, followed by staining with SYTO 9/PI for 30 min. The live/dead bacteria in the 3D structure of the biofilms were subsequently captured via a confocal laser scanning microscope. Mature biofilms were also incubated on silicon wafers in confocal dishes and treated with PBS or Cu_x_Te nanozymes for 4 h. They were then fixed with 2.5% glutaraldehyde, gradient dehydrated with different concentrations of ethanol, and finally observed via a scanning electron microscope to assess the biofilm morphology of each group. In a 24-well plate, mature biofilms were cultivated and then treated with Cu_x_Te nanozymes at a concentration of 30 μg/ml for 12 h to conduct a biofilm disruption experiment. For the biofilm inhibition experiment, suspensions of *E. coli* and *P. aeruginosa* (1 × 10^8^ CFU/ml) were added to 900 μl of LB medium containing Cu_x_Te nanozymes (30 μg/ml) and incubated statically at 37 °C for 48 h. After the aforementioned treatments were performed in a 24-well plate, the supernatant was discarded, and the residual suspended bacteria at the bottom were gently washed with PBS. This process was repeated 3 times. The biofilms at the bottom were stained with crystal violet dye for 30 min, photographed, and then decolorized with absolute ethanol. The absorbance at a peak wavelength of 570 nm was measured via a microplate reader.

### In vivo antibacterial activity and wound healing

The *P. aeruginosa*-infected burn wound model employs 6- to 8-week-old male BALB/c mice. The mice were randomly divided into 4 groups: PBS (control), CG, Cu_x_Te, and Cu_x_Te@CG. The mice were anesthetized with 1% pentobarbital sodium (40 mg/kg), and the hair on their backs was shaved. A custom-made burn apparatus with a burn diameter of 8 mm was then used to burn the backs of the mice at 80 °C for 8 s. Within 1 h post-burn, the mice were intraperitoneally injected with physiological saline. Specifically, the burn area accounts for 10% of the total body surface area of the mice, necessitating an injection of 1.6 ml of physiological saline for a 25-g mouse. This is achieved by doubling the concentration of the fluid volume required for the percentage of body surface area per the Parkland formula, which typically recommends an injection of 4 ml of fluid per kilogram of body weight to replenish the fluid lost after a burn. After 24 h, surgical scissors were used to create an approximately 8-mm burn wound by cutting along the burned tissue area on the back. Subsequently, 30 μl (1×10^9^ CFU/ml) of *P. aeruginosa* suspension was inoculated onto the wound and allowed to air dry. The mice were then randomly divided into 4 groups for treatment, with PBS, CG, Cu_x_Te, and Cu_x_Te@CG being applied topically to the wounds. The progress of wound healing was documented daily via a digital camera. On the 10th day, after photographic documentation, the mice were anesthetized and euthanized, and their skin samples were collected. For the calculation of the wound-healing rate, the specific formula applied was as follows: Wound healing rate (%) = [(Original wound area − Residual wound area at each time point)/Original wound area] × 100. Wound healing was analyzed through Masson and H&E staining, and changes in inflammation among the groups were assessed via immunofluorescence staining. The hearts, livers, spleens, lungs, and kidneys of the mice were subjected to H&E staining, and blood samples were collected for serum biochemistry/whole blood tests.

### Transcriptomic analysis

All specimens were prepared in compliance with the guidelines provided by Personal Technology Co., Ltd. (Shanghai, China). In addition, data processing, including correlation analysis, DEG analysis, GO analysis, and KEGG analysis, was performed on a personal technology cloud platform (https://www.genescloud.cn/home).

### Cells and bacteria

The gram-negative bacteria used were *E. coli* (*DH5α*), *P. aeruginosa* (*ATCC9027*), and *K. pneumoniae* (*ATCC13883*), and the gram-positive bacteria used were *S. aureus* (*NCTC 8325*), MRSA (*MU50*), and *E. faecalis* (*ATCC29212*). A mouse fibroblast line (L929 cells) (SCSP-5039) was obtained from the Chinese Academy of Science.

### Statistical analysis

At least 3 independent experiments were performed for each condition unless otherwise specified. For multiple comparisons, 1-way/2-way analysis of variance (ANOVA) accompanied by Tukey’s post hoc test for significant differences was performed, wherein a *P* value below 0.05 was deemed indicative of a significant difference, and the data are indicated with *P < 0.05, **P < 0.01, and ***P < 0.001.

## Ethical Approval

The animal-related procedures were carried out in conformity with the guidelines established by the Institutional Animal Care and Use Committee of Anhui Medical University (approval number: No. LLSC20242210). All mice were housed and euthanized in compliance with the animal research policies of the National Ministry of Health as per standard regulations.

## Data Availability

The datasets underpinning the results of this research can be obtained from the corresponding authors upon a justified request.
