# Supplementary material for: Engineering a Gram-Negative Bactericidal Hydrogel: CuxTe Nanozyme Functions as a Specific Killer by Hijacking LPS and Flagella Biosynthesis
Source: Research (Wash D C). 2026 Feb 25;9:1140. doi: 10.34133/research.1140 (PMC12932864; doi:10.34133/research.1140)
Supplement: Supplementary 1 — Figs. S1 to S12 Table S1 [file research.1140.f1.doc]

**Engineering a Gram-Negative Bactericidal Hydrogel: Cu_x_Te Nanozyme Functions as a Specific Killer by Hijacking LPS and Flagella Biosynthesis**

*Jianguo Niu^1^, Yuhao Xue^1^, Wenqi Wang^1^, Wei Zhang^1^, Min Wang^1^, Jiaqi Qin^1^, Dongliang Yang^2*^, Xianwen Wang ^1*^*

1. School of Biomedical Engineering, Anhui Medical University, Hefei 230022, P. R. China.
2. School of Physical and Mathematical Sciences, Nanjing Tech University, Nanjing 211816, P. R. China

***Corresponding Authors:** E-mail: yangdl1023@njtech.edu.cn (*D. Yang*); xianwenwang@ahmu.edu.cn (*X. Wang*)

**This supplementary information includes the following:**

1. Supplementary Figures

2. Supplementary table

**1. Supplementary Figures**


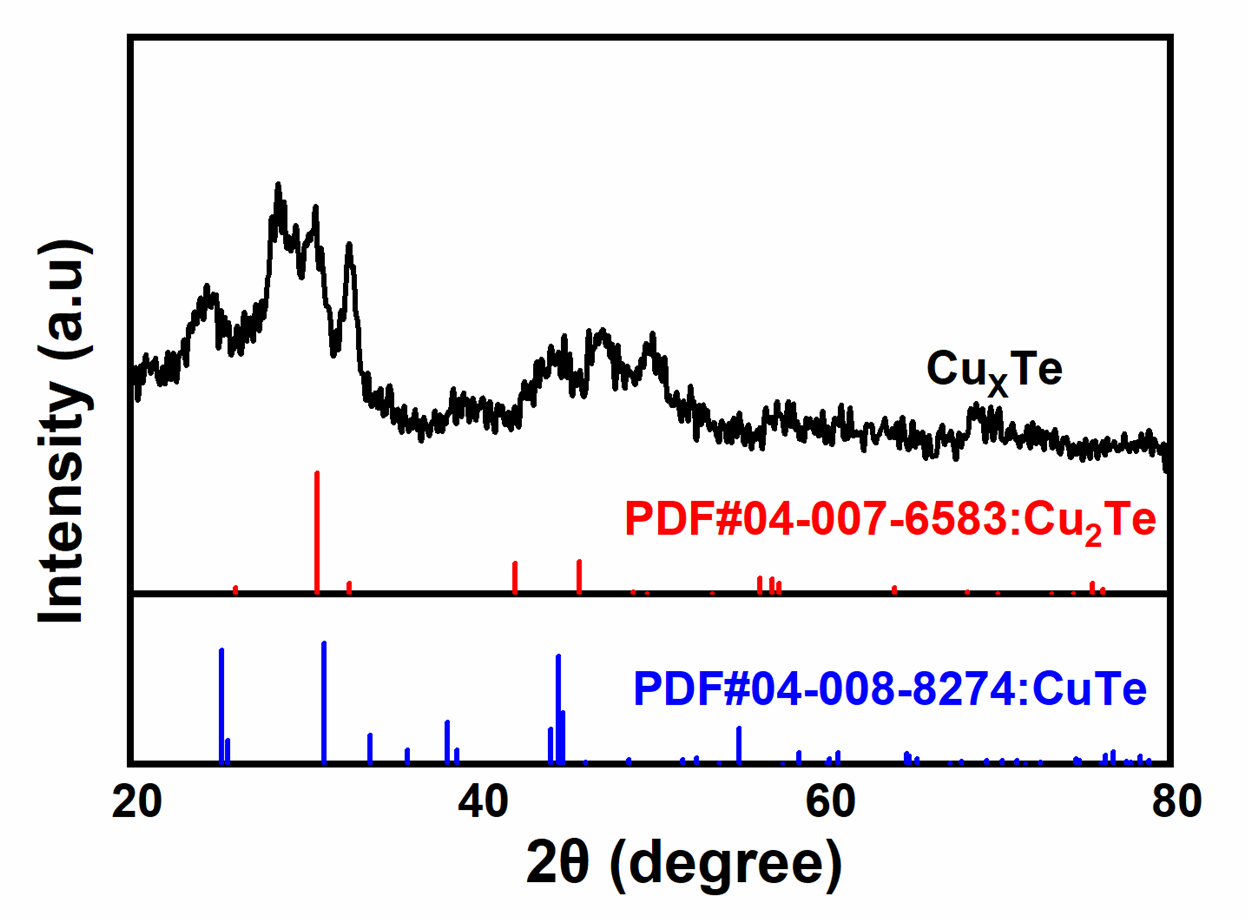


**Figure S1.** X-ray powder diffraction (XRD) patterns of Cu_x_Te nanozymes.


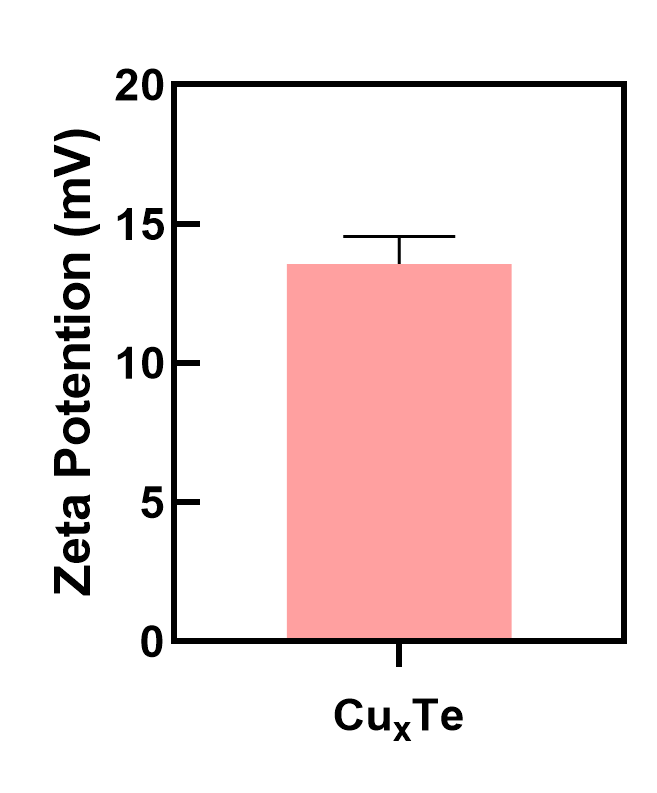


**Figure S2.** Zeta potential values of Cu_x_Te nanozymes.


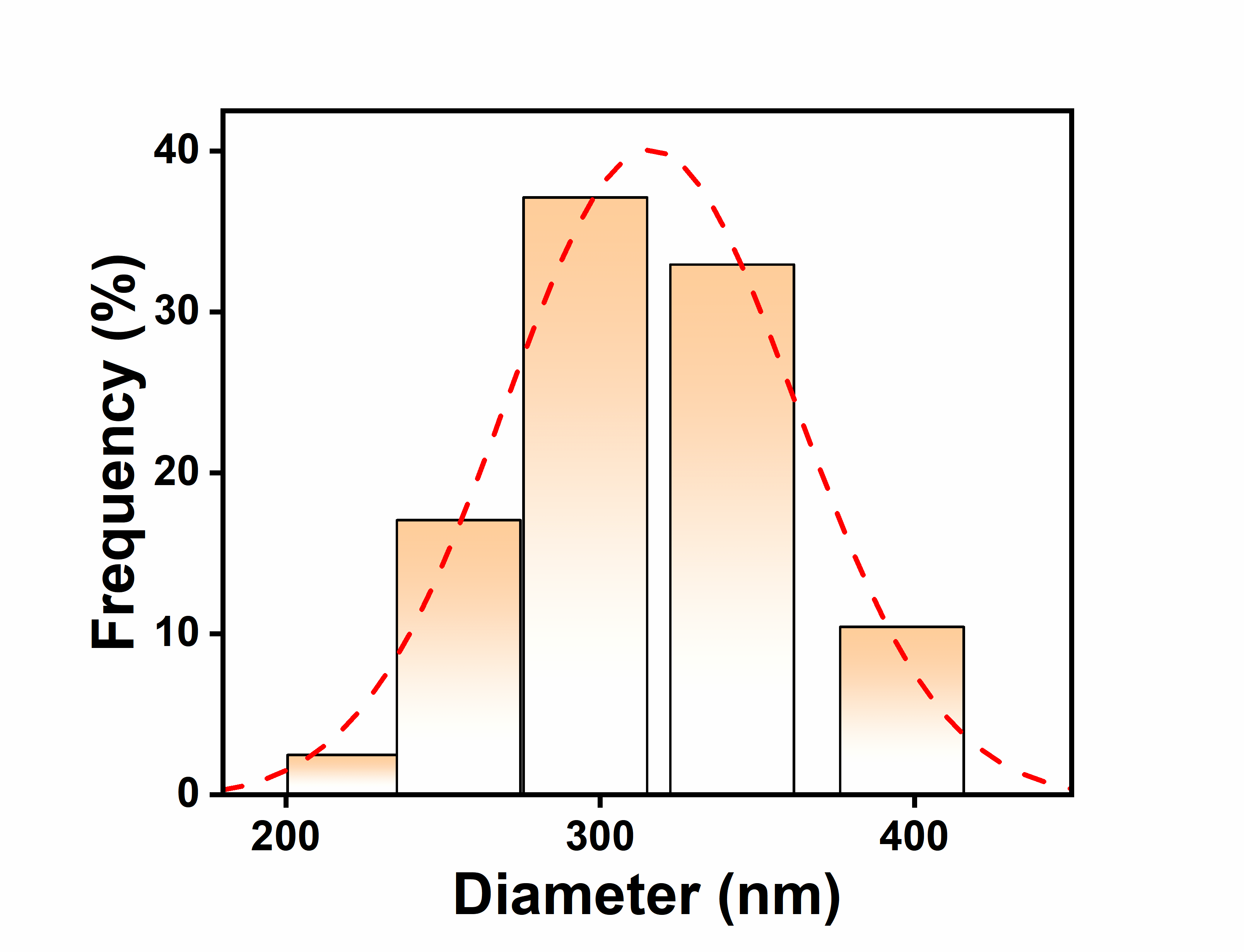
**Figure S3.** Particle diameter values of Cu_x_Te nanozymes.


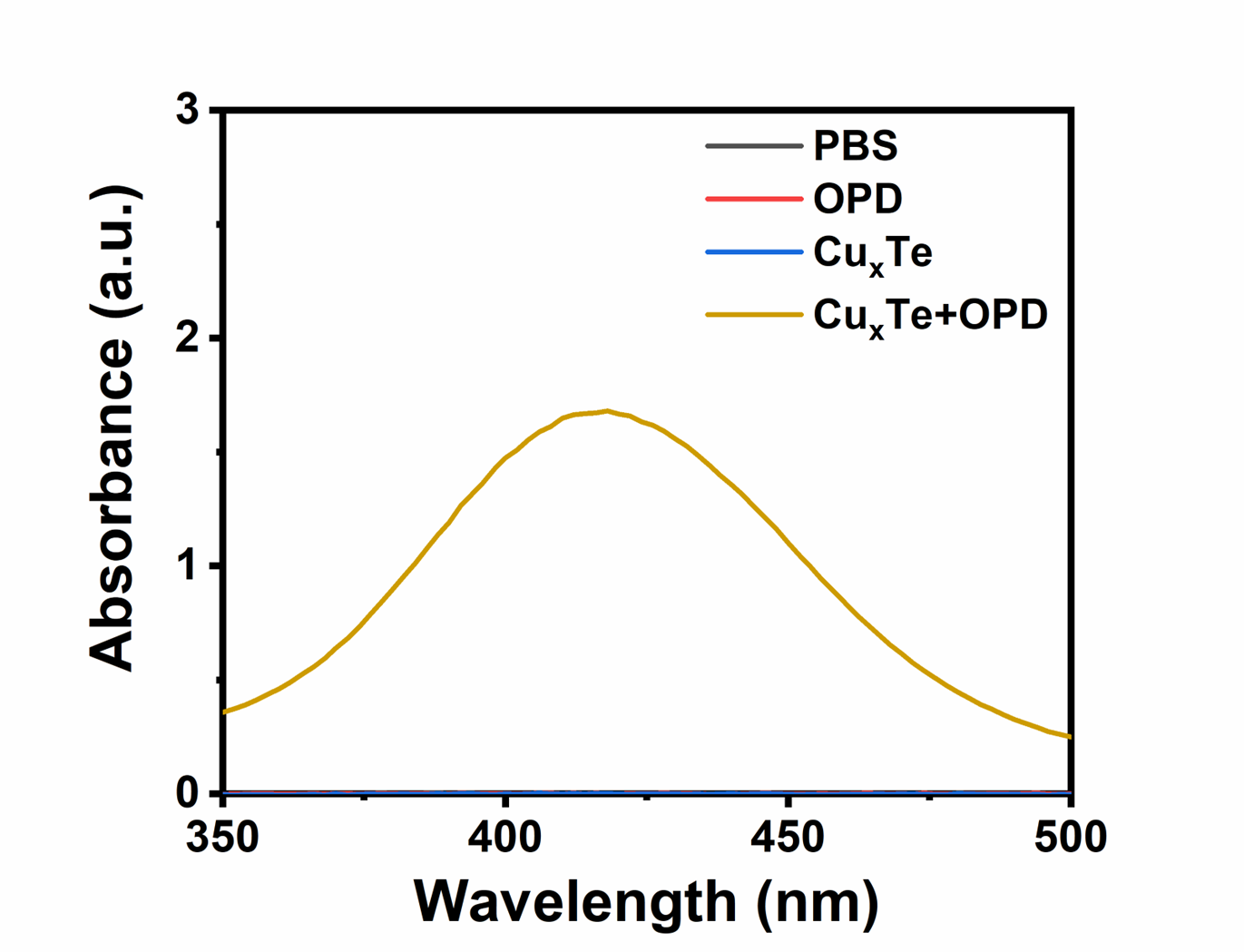


**Figure S4.** The absorbance of different groups after the addition of OPD.


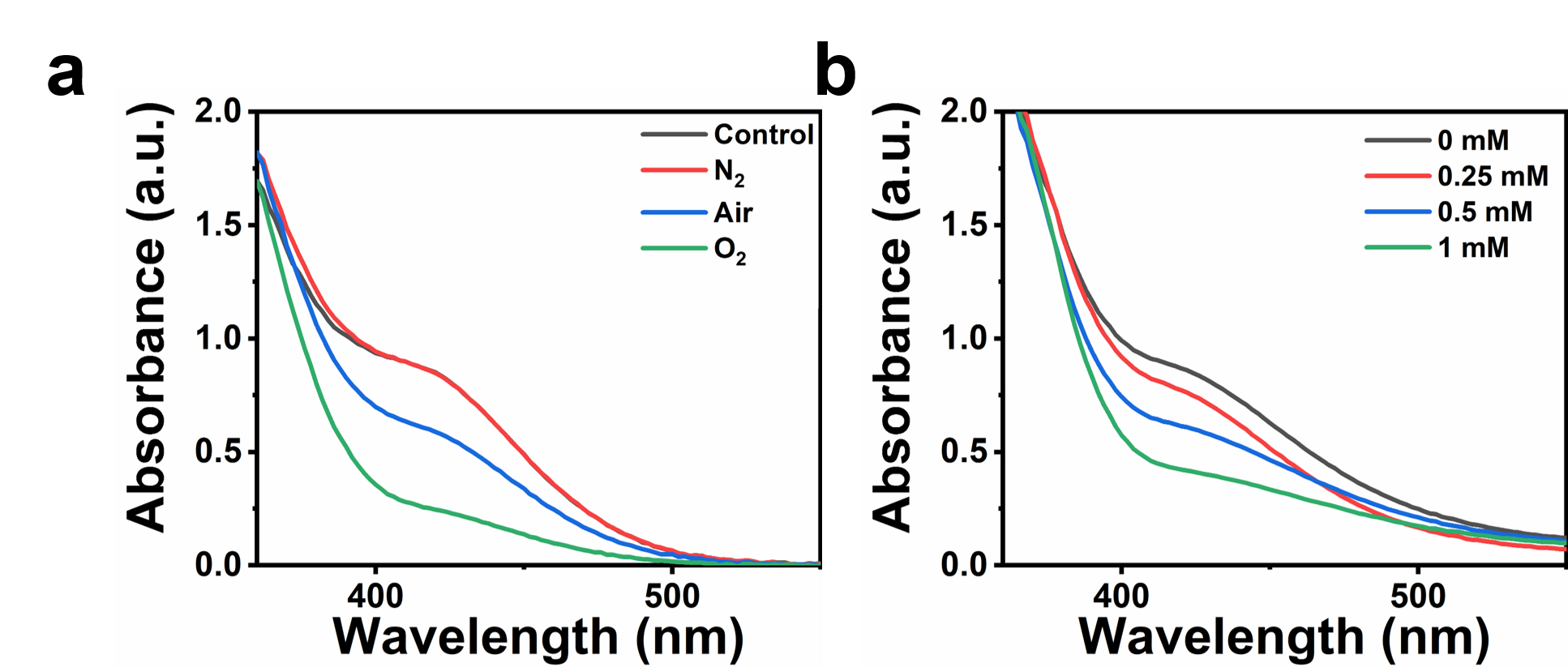
**Figure S5. a)** Detection of GSH clearance activity of Cu_x_Te nanozymes under varying oxygen concentration conditions. **b)** Detection of GSH clearance activity of Cu_x_Te under varying H_2_O_2_ concentration conditions.


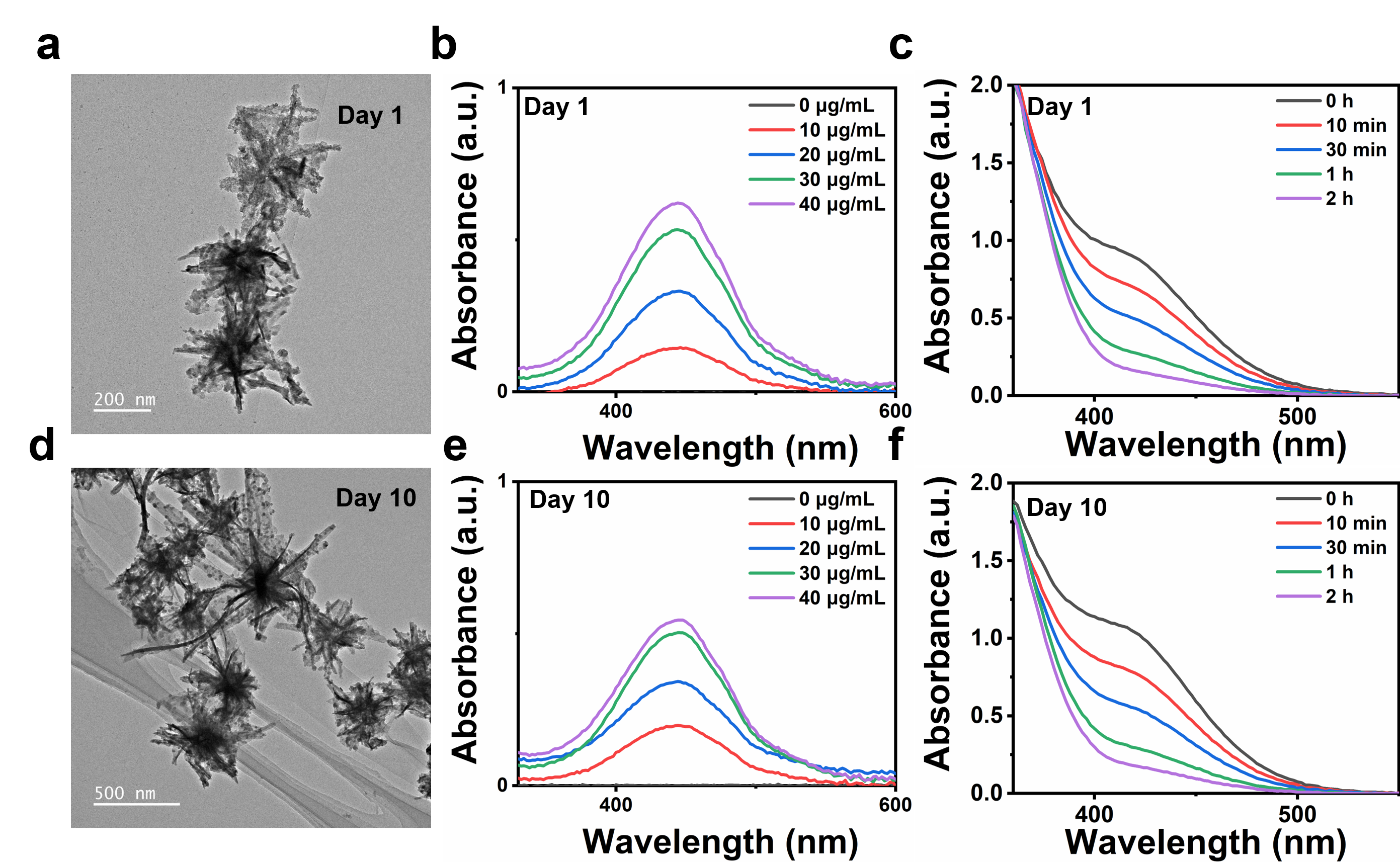


**Figure S6.** **a, d)** TEM images of Cu_x_Te nanozymes in aqueous solution on Day 1 and Day10. **b, e)** Absorbance of different concentration of Cu_x_Te nanozymes after the addition of OPD on Day 1 and Day10. **c, f)** Time-dependent GSH depletion of Cu_x_Te nanozymes at different concentrations using the DTNB probe on Day 1 and Day10.


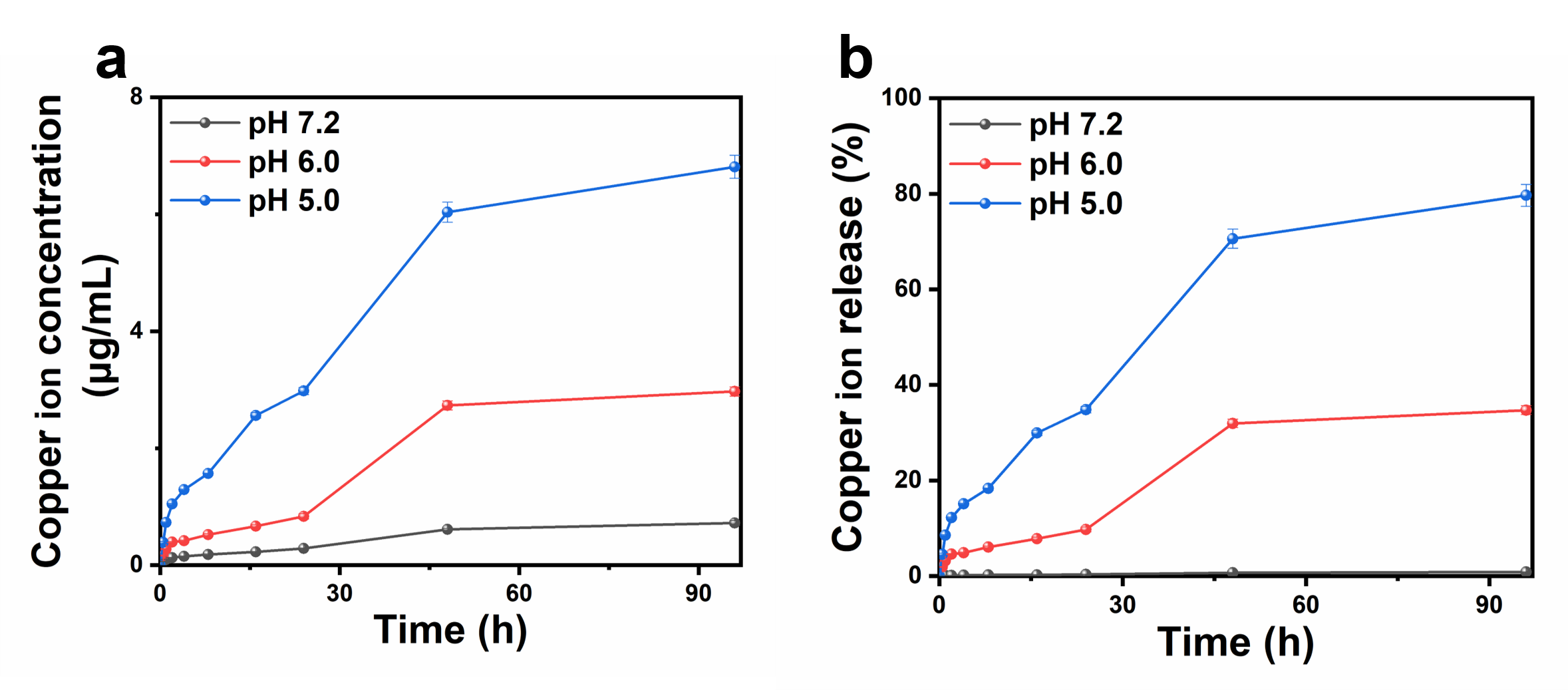
**Figure S7. a)** Copper ion concentration of Cu_x_Te nanozymes at different pH values and time points. **b)** Copper ion release of Cu_x_Te nanozymes at different pH values and time points.


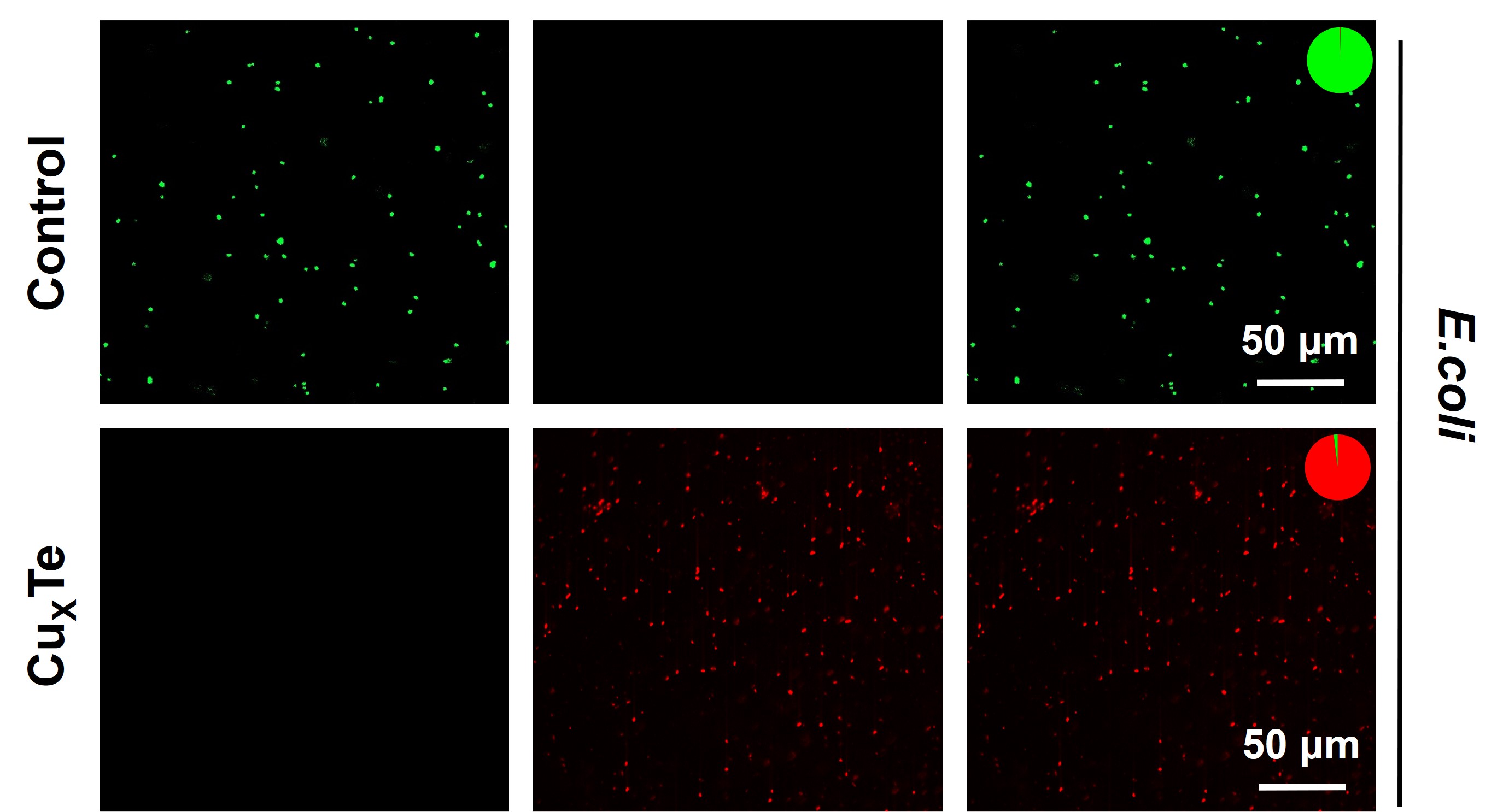


**Figure S8.** Fluorescence images of dead (red) and live (green) bacteria of *E. coli* in different treatment groups.


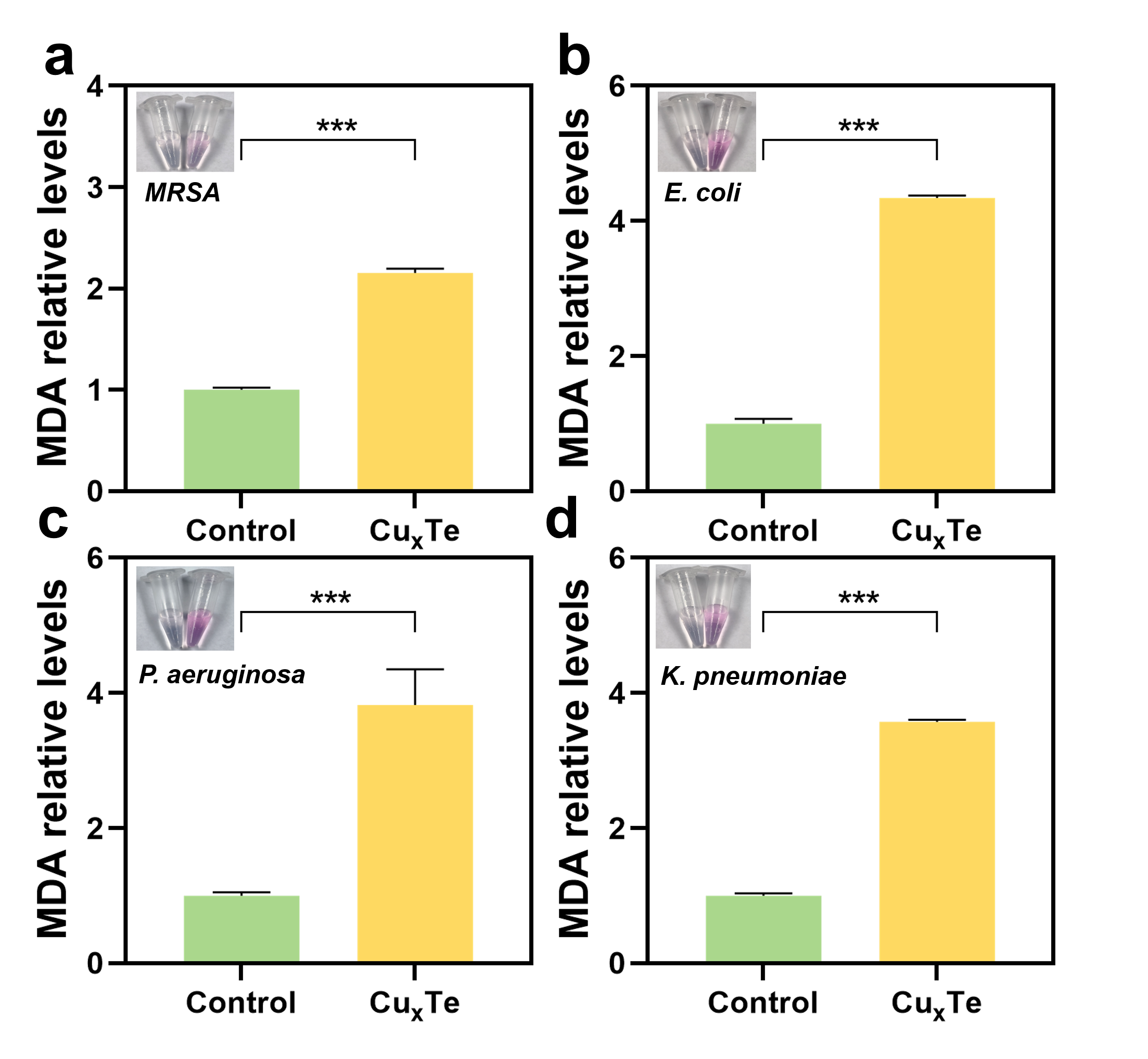
**Figure S9. a)** MDA relative levels in *MRSA* under different treatment groups **b)** MDA relative levels in *E. coli* under different treatment groups. **c)** MDA relative levels in *P. aeruginosa* under different treatment groups. **d)** MDA relative levels in *K. pneumoniae* under different treatment groups. Statistical analysis was performed using one-way ANOVA followed by Tukey’s post hoc test. (*p<0.05, **p<0.01 and ***p<0.001) (mean ± SD, n = 3).


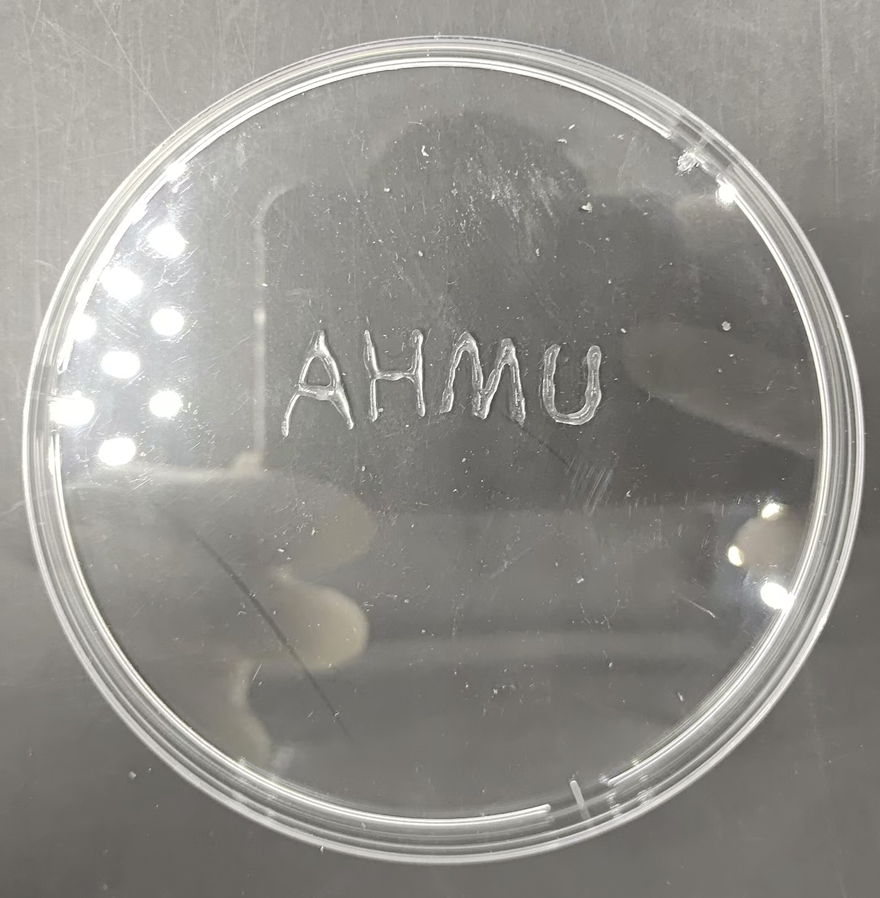


**Figure S10.** The Cu_x_Te@CG hydrogel was injected via a syringe to form the letters "AHMU".


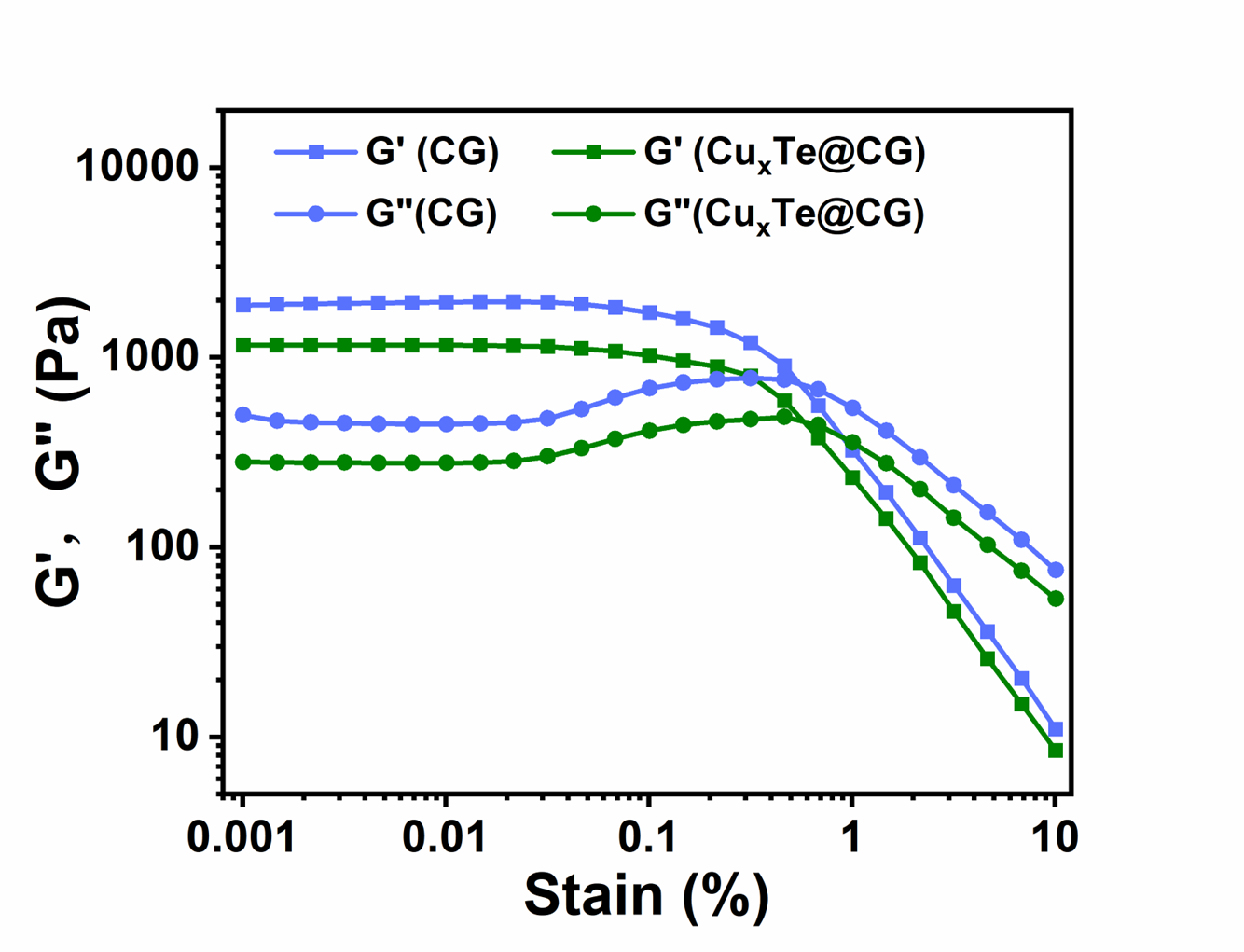


**Figure S11.** Rheological properties of the Cu_x_Te@CG hydrogel: Strain sweep.


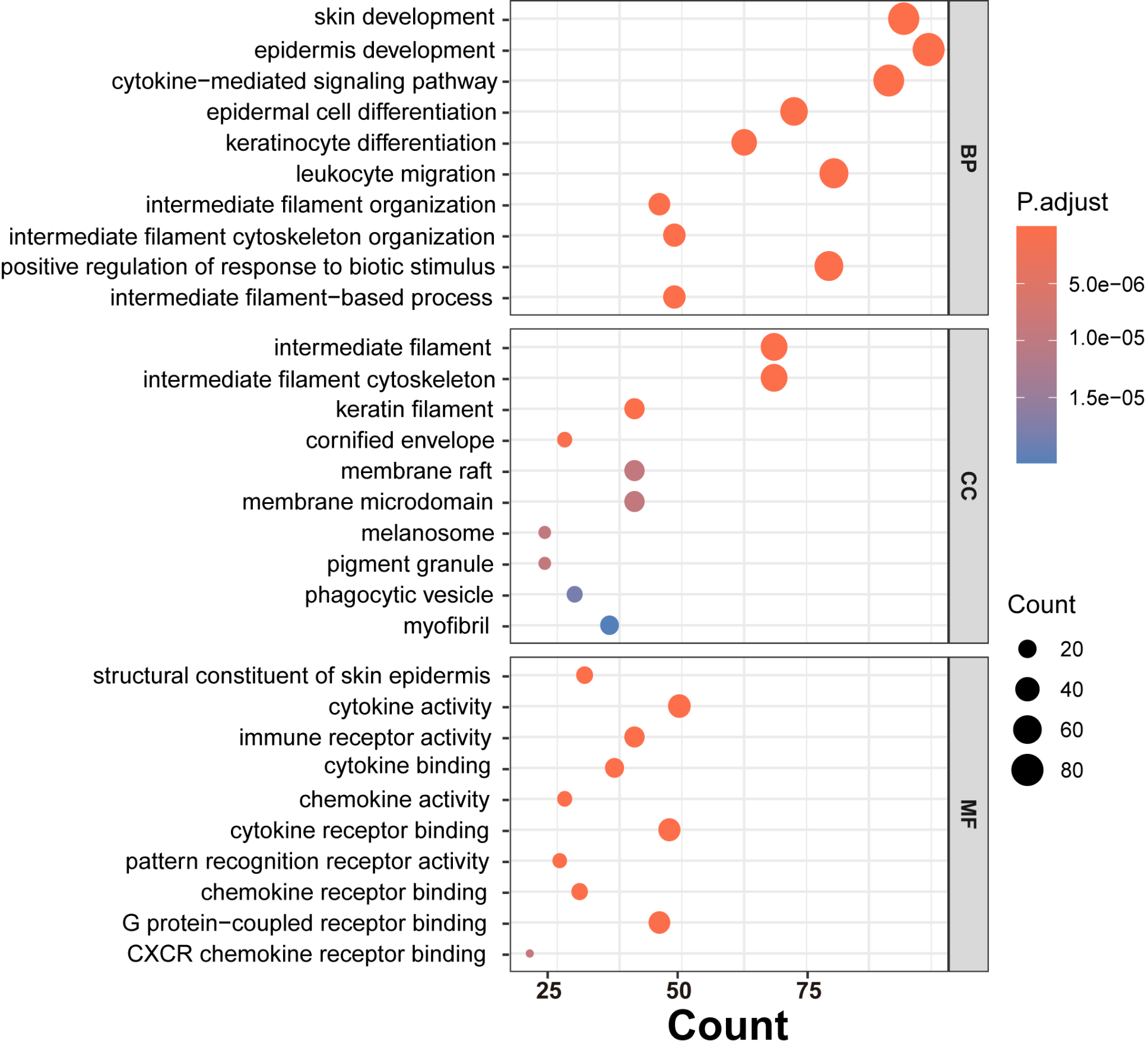


**Figure S12.** Genetic analysis of GO genes in burn wound tissue *in vivo.*

1. **Supplementary table**

**Table S1.** RT‒qPCR primers for *P. aeruginosa.*

| **Ptahway ID** | **Primer** | **Sequence (5’ — 3’)** |  |
| --- | --- | --- | --- |
| *lpxD* | Forward | CACTGTCCGATACGCTGAT |  |
| *lpxD* | Reverse | GCCGATCTGCACGTTGTG |  |
| *rfaD* | Forward | AGAACAACTACCGCTACAG |  |
| *rfaD* | Reverse | GACGAGGCGTAGAGGAAC |  |
| *pagL* | Forward | GGCGATCCACTATTCCAA |  |
| *pagL* | Reverse | ATCTTGTAGAACAGGCTGTA |  |
| *eptA* | Forward | GCCTACTTCAAGCGCTATCC |  |
| *eptA* | Reverse | CGGGGTGAACTTCTCGTACT |  |
| *flgC* | Forward | CCGACATGATTTCCGCCAG |  |
| *flgC* | Reverse | TCTGCATCATCTGTTTGGCG |  |
| *fliaA* | Forward | TGGAGTGCGTATGTATAGC |  |
| *fliaA* | Reverse | TTGACCAGTGGTGCGTAA |  |
| *lon* | Forward | CGGAGCAGAACCACAACTTC |  |
| *lon* | Reverse | GTGCAGAGGAACATCACGTC |  |
| *rpoS* | Forward | CACTTCCTTCTCTTCCAA |  |
| *rpoS* | Reverse | CGATTTCGTTGAGATACAG |  |
| *algP* | Forward | CGCTGGTCGATTCGGAAA |  |
| *algP* | Reverse | CTTGGTGCGAGCCTTGTG |  |
| *tonB1* | Forward | GAACTCAACCTTGGCCACG |  |
| *tonB1* | Reverse | CGAGCTGGACGAAATTCACC |  |
